# Antifungal Activity of *Hedychium spicatum* Essential Oil Against Candidemia‐Causing *Candida tropicalis* and *Candida parapsilosis*: In Vitro Evaluation and Computational Identification of Cis‐γ‐Bisabolene as a Putative CYP51‐Binding Constituent

**DOI:** 10.1002/cbdv.71742

**Published:** 2026-09-27

**Authors:** Preeti Verma, Devesh Singh Baral, Rakesh Verma

**Affiliations:** ^1^ Department of Chemistry PG College Kapkot SSJ University Almora Uttarakhand India; ^2^ Department of Zoology SSJ University L.S.M. Campus Pithoragarh Uttarakhand India

**Keywords:** *Hedychium spicatum*, CYP51 inhibition, ergosterol biosynthesis pathway, essential oil, zone of inhibition

## Abstract

Candidiasis is caused by *Candida parapsilosis* and *Candida tropicalis* emerges due to antifungal resistance and limited therapies. We aim to assess the antifungal activity of *Hedychium spicatum* essential oil (HSEO), followed by computational analyses to identify potential lead phytochemicals, targeting lanosterol 14α‐demethylase (CYP51), a key enzyme in ergosterol biosynthesis. GC‐MS identified 29 phytoconstituents, with τ‐cadinol (17.94%) as the major component. Disc diffusion demonstrated concentration‐dependent antifungal activity against *C. parapsilosis* and *C. tropicalis*, with inhibition zones of 11.00 ± 0.53 and 10.40 ± 0.36 mm, respectively, at 100% (v/v). Homology models of CYP51 exhibited structural reliability based on Ramachandran and validation metrics. Molecular docking identified cis‐γ‐bisabolene with the lowest binding energies (−7.6 and −7.9 kcal/mol) against haem‐free CYP51 models of *C. parapsilosis* and *C. tropicalis*, respectively, through hydrophobic interactions. ADMET predictions indicated favorable oral absorption, Lipinski compliance, minimal CYP450 inhibition risk, and no hepatotoxicity or mutagenicity. MD simulations confirmed stable complexes using RMSD, RMSF, Rg, and SASA profiles. MM‐PBSA showed favorable binding energies of −6.02 and −8.05 kJ/mol compared to fluconazole (0.98 and 0.66 kJ/mol). DFT showed comparable HOMO–LUMO gaps (6.216 and 6.176 eV) and global hardness (3.108 and 3.088 eV). Overall, HSEO shows promising antifungal potential, with cis‐γ‐bisabolene as a lead candidate.

## Graphical abstract

*Hedychium spicatum* essential oil (HSEO) exhibited concentration‐dependent antifungal activity against *Candida parapsilosis* and *Candida tropicalis*. GC‐MS identified 29 phytoconstituents, with *τ*‐cadinol as the major component. Computational analysis identified cis‐γ‐bisabolene as a promising CYP51 inhibitor, showing favorable binding, ADMET properties, molecular stability, MM‐PBSA, and DFT profiles, supporting its potential as an antifungal lead.

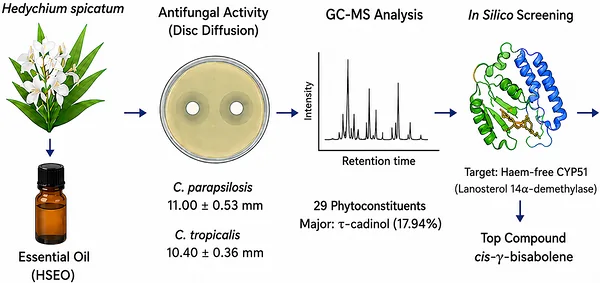

**Abbreviations**
DFT: Density functional theory
HOMO: Highest occupied molecular orbital
LUMO: Lowest unoccupied molecular orbital
MDS: Molecular dynamics simulation
MM/PBSA: Molecular mechanics Poisson–Boltzmann surface area
RMSD: Root mean square deviation
RMSF: Root mean square fluctuation
SASA: Solvent accessible surface area

## 1 Introduction

Invasive fungal infections represent a significant public health concern, substantially increasing morbidity and mortality, particularly among hospitalized and immunocompromised individuals [1]. Among these, candidiasis caused by *Candida* spp. remains one of the most prevalent and potentially life‐threatening mycoses worldwide [2]. Although *Candida albicans* remains the most common species, the prevalence of other *Candida* species, particularly *Candida parapsilosis* and *Candida tropicalis*, has risen significantly; these species are now increasingly associated with invasive candidiasis worldwide [3]*. Candida parapsilosis* is a prominent cause of catheter‐associated bloodstream infections, largely due to its robust biofilm‐forming capacity on indwelling medical devices, and is often linked to neonatal sepsis and infective endocarditis [4]. *Candida tropicalis* is predominantly reported in immunocompromised individuals, especially patients with haematological malignancies and neutropenia, and is known for causing disseminated infections [5]. In addition to bloodstream infections, both species contribute to a wide range of mucosal and systemic manifestations, including oral candidiasis, vulvovaginal candidiasis, and urinary tract infections, especially in immunocompromised patients [6, 7]. The rising incidence and evolving antifungal resistance among these non‐*C. albicans* species (NCAC) highlight the pressing need to explore alternative natural therapeutic interventions.

Lanosterol 14α‐demethylase (CYP51), an essential enzyme involved in ergosterol biosynthesis in fungi, represents a major molecular target in antifungal treatment strategies [8]. The enzyme belongs to the cytochrome P450 superfamily, a large and highly conserved group of heme‐containing monooxygenases [9]. Lanosterol serves as the substrate for CYP51, which mediates the oxidative demethylation of the 14α‐methyl group, a critical step in ergosterol biosynthesis required for preserving fungal membrane integrity [10]. Ergosterol plays a crucial role in maintaining membrane fluidity, structural integrity, membrane dynamics, and cellular responses to environmental stress [11]. Due to its central role in ergosterol biosynthesis and fungal viability, CYP51 serves as the primary molecular target of azole antifungal agents, including fluconazole. Inhibition of this enzyme disrupts cell membrane integrity, ultimately resulting in growth arrest and fungal cell death [10, 12].

Medicinal plants have long been recognized as rich sources of therapeutic compounds, and their bioactive constituents continue to provide promising strategies for the control of diverse pathogenic microorganisms. Studies on the plant *Hedychium spicatum* Sm., a medicinally important species of the Zingiberaceae family, which is traditionally referred to as Shati (Sanskrit) and is popularly known as Van Haldi or Kapoor Kachari in Hindi [13]. *H. spicatum* is a rhizomatous perennial herb that grows up to 1–2 m in height, characterized by long oblong‐lanceolate green leaves and dense terminal spikes of orange‐white hermaphroditic flowers [14]. The plant *H. spicatum* has been traditionally employed for the management of diabetes due to its reported antidiabetic activity [15]. The rhizomes of the plant are a reservoir of bioactive constituents, including essential oils, diarylheptanoids, starch, resins, organic acids, glycosides, albumen, and saccharides, has been historically recommended for blood purification [15]. Essential oils are volatile secondary metabolites of plant origin that contribute to defence mechanisms against pathogens and herbivores, while also participating in pollinator attraction and broader ecological signaling processes [16]. present study was undertaken to assess the antifungal efficacy of the essential oil using in vitro disc diffusion assays, followed by computational investigations to explore its molecular mechanism of action. The antifungal activity of the essential oil was preliminarily assessed using the disc diffusion assay by measuring the zone of inhibition, a widely accepted screening method for evaluating antimicrobial efficacy [17]. Advances in computational methodologies, particularly molecular docking, have significantly contributed to antifungal drug discovery by facilitating the prediction and analysis of interactions between bioactive ligands and defined molecular targets [18]. These computational approaches provide valuable mechanistic insights into ligand–enzyme interactions and enable the validation of potential inhibitory effects at the molecular level prior to extensive experimental investigations.

Despite the reported medicinal and antimicrobial potential of *H. spicatum*, limited information is available on the antifungal activity of its essential oil against *Candida* species and its underlying molecular basis. The potential interactions of its essential oil constituents with CYP51 of *C. parapsilosis* and *C. tropicalis* also remain insufficiently explored. We hypothesized that *H. spicatum* essential oil may exhibit antifungal activity against these species and interact with CYP51. Therefore, this study aimed to characterize the essential oil by GC‐MS, evaluate its preliminary antifungal activity against *C. parapsilosis* and *C. tropicalis*, and investigate potential CYP51–ligand interactions using molecular docking, ADMET prediction, molecular dynamics, MM‐PBSA, and DFT. The novelty of this study lies in integrating experimental evaluation with multi‐level computational analysis against CYP51 from two clinically relevant *Candida* species. These findings will provide preliminary evidence for potential CYP51‐ligand interactions and identify constituents for further experimental investigation.

## 2 Results

The findings obtained from the experimental and computational analyses are presented below.

### 2.1 GC and GC‐MS

**FIGURE 1:**
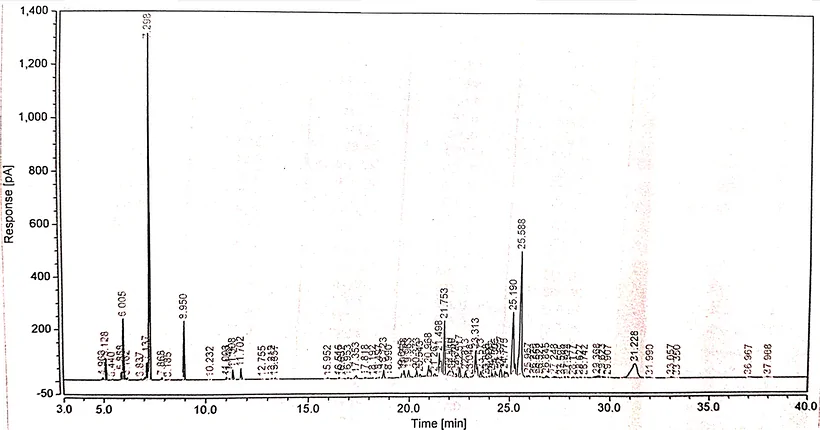
GC2‐13MS chromatogram of HSEO, with selected identified phytoconstituents indicated. The *x*‐axis represents retention time (min), and the *y*‐axis represents detector response (pA).

GC‐MS analysis of the essential oil revealed retention times ranging from 5.133 min for α‐pinene to 33.427 min for 8‐cedren‐13‐ol, reflecting the chemical diversity of the volatile constituents (Figures 1 and S1, and Table S1). A total of 29 compounds were identified based on their retention index and mass spectral comparison with standard libraries. *τ*‐Cadinol (17.94%) was the predominant constituent, followed by 1,8‐cineole (17.54%). Other notable components included γ‐eudesmol (8.26%), germacra 4(15),5,10(14)‐trien‐1‐α‐ol (7.96%), cis‐γ‐bisabolene (5.24%), linalool (4.23%), sabinene (3.38%), germacrene D (1.94%), and *δ*‐cadinene (1.92%), while the remaining constituents were present in lower proportions and are listed in Table 1. The identified compounds were classified into four major chemical groups: oxygenated monoterpenes (OM), oxygenated sesquiterpenes (OS), monoterpene hydrocarbons (MH), and sesquiterpene hydrocarbons (SH). OS constituted the dominant fraction of the oil, followed by OM, SH, and MH. In terms of distribution, 11 compounds belonged to OS, 7 to SH, 6 to OM, and 5 to MH. Among these, 1,8‐cineole (17.54%) was the most abundant OM constituent, sabinene (3.38%) was the predominant MH, *τ*‐cadinol (17.94%) was the major OS compound, and cis‐γ‐bisabolene (5.24%) represented the most abundant SH. The molecular weights of the detected constituents ranged from 136.23 g/mol (limonene) to 222.37 g/mol (cubebol, elemol, ledol, α‐cadinol, α‐eudesmol, γ‐eudesmol, and τ‐cadinol). The number of rotatable bonds varied from 0 to 4 (maximum observed in linalool), while one hydrogen‐bond acceptor and one hydrogen‐bond donor were observed among the compounds. The topological polar surface area (TPSA) ranged from 0 to 20.2 Å^2^. These parameters indicate limited molecular flexibility and moderate hydrogen‐bonding capacity, suggesting favorable membrane permeability and potential target interaction. The detailed physicochemical properties of all identified compounds are summarized in Table 2.

**TABLE 1:** GC‐MS analysis of HSEO showing the identified chemical constituents, their retention times, calculated retention indices, literature retention indices, and relative abundance.

S. no.	Compound	Class composition	RT* (Min)	RI^a^, *	RI^b^, *	% of compound
1	1,8‐cineole	OM	7.453	1038	1031	17.54
2	14‐hydroxy‐(Z) Caryophyllene	OS	29.357	1670	1667	0.66
3	8‐Cedren‐13‐ol	OS	33.427	1690	1689	0.19
4	cis‐Sabinene hydrate	OM	8.728	1074	1070	0.03
5	cis‐γ‐Bisabolene	SH	21.762	1517	1515	5.24
6	Cubebol	OS	22.578	1517	1515	0.95
7	Elemol	OS	24.662	1555	1549	1.20
8	Germacra 4(15),5,10(14) trien‐1‐α‐ol	OS	32.012	1687	1686	7.96
9	Germacrene D	SH	20.97	1481	1481	1.94
10	Isolongifelene	SH	18.718	1396	1390	0.81
11	Ledol	OS	24.922	1608	1602	1.20
12	Limonene	MH	6.852	1034	1029	1.26
13	Linalool	OM	9.043	1101	1096	4.23
14	Sabinene	MH	6.013	978	975	3.38
15	Terpinen‐4‐ol	OM	11.33	1180	1177	0.77
16	Trans‐Sabinene hydrate	OM	11.012	1104	1098	0.03
17	α‐Cadinol	OS	28.453	1655	1654	0.10
18	α‐Eudesmol	OS	27.922	1653	1653	0.13
19	α‐Pinene	MH	5.133	941	939	1.05
20	α‐Terpineol	OM	11.75	1192	1188	0.86
21	β‐Acoradiene	SH	19.96	1473	1470	0.70
22	β‐Caryophyllene	SH	19.652	1418	1408	0.77
23	β‐Guaiene	SH	21.603	1495	1493	1.09
24	β‐Pinene	MH	6.238	982	979	0.13
25	γ‐Eudesmol	OS	25.233	1637	1632	8.26
26	γ‐Muurolene	SH	20.543	1479	1479	0.30
27	γ‐Terpinene	MH	7.888	1063	1059	0.15
28	δ‐Cadinene	OS	23.355	1525	1523	1.92
29	τ‐Cadinol	OS	25.693	1645	1640	17.94

Abbreviations: MH, monoterpene hydrocarbon; OM, oxygenated monoterpene; SH, sesquiterpene hydrocarbon; OS, oxygenated sesquiterpene.

^*^ RT: Retention time.

^a^ RI: Experimental retention indices (calculated).

^b^ RI: Retention indices according to literature (Adams, 2017).

**TABLE 2:** Physicochemical properties of the compounds identified by GC‐MS analysis of *H. spicatum* essential oil.

S. no.	Compound	PubChem ID	MF*	MW* (g/mol)	nRB*	HBD*	HBA*	TPSA* (Å^2^)
1	1,8‐cineole	2758	C_10_H_18_O	154.25	0	0	1	9.2
2	14‐hydroxy‐(Z) Caryophyllene	12300116	C_15_H_24_O	220.35	1	1	1	20.2
3	8‐Cedren‐13‐ol	519545	C_15_H_24_O	220.35	1	1	1	20.2
4	cis‐Sabinene hydrate	11744854	C_10_H_18_O	154.25	1	1	1	20.2
5	cis‐γ‐Bisabolene	3033866	C_15_H_24_	204.35	3	0	0	0
6	Cubebol	11276107	C_15_H_26_O	222.37	1	1	1	20.2
7	Elemol	92138	C_15_H_26_O	222.37	3	1	1	20.2
8	Germacra 4(15),5,10(14) trien‐1‐α‐ol	13304977	C_15_H_24_O	220.35	1	1	1	20.2
9	Germacrene D	5317570	C_15_H_24_	204.35	1	0	0	0
10	Isolongifelene	11127402	C_15_H_24_	204.35	0	0	0	0
11	Ledol	11074994	C_15_H_26_O	222.37	0	1	1	20.2
12	Limonene	22311	C_10_H_16_	136.23	1	0	0	0
13	Linalool	6549	C_10_H_18_O	154.25	4	1	1	20.2
14	Sabinene	18818	C_10_H_16_	136.23	1	0	0	0
15	Terpinen‐4‐ol	11230	C_10_H_18_O	154.25	1	1	1	20.2
16	Trans‐Sabinene hydrate	6431628	C_10_H_18_O	154.25	1	1	1	20.2
17	α‐Cadinol	10398656	C_15_H_26_O	222.37	1	1	1	20.2
18	α‐Eudesmol	92762	C_15_H_26_O	222.37	1	1	1	20.2
19	α‐Pinene	6654	C_10_H_16_	136.23	0	0	0	0
20	α‐Terpineol	17100	C_10_H_18_O	154.25	1	1	1	20.2
21	β‐Acoradiene	20055537	C_15_H_24_	204.35	1	0	0	0
22	β‐Caryophyllene	5281515	C_15_H_24_	204.35	0	0	0	0
23	β‐Guaiene	15560252	C_15_H_24_	204.35	0	0	0	0
24	β‐Pinene	14896	C_10_H_16_	136.23	0	0	0	0
25	γ‐Eudesmol	6432005	C_15_H_26_O	222.37	1	1	1	20.2
26	γ‐Muurolene	6432308	C_15_H_24_	204.35	1	0	0	0
27	γ‐Terpinene	7461	C_10_H_16_	136.23	1	0	0	0
28	δ‐Cadinene	441005	C_15_H_24_	204.35	1	0	0	0
29	τ‐Cadinol	160799	C_15_H_26_O	222.37	1	1	1	20.2

*MF: Molecular Formula; MW: Molecular Weight; nRB: No. of Rotatable Bonds; HBD: Hydrogen Bond Donor; HBA: Hydrogen Bond Acceptor; TPSA: Topological Polar Surface Area.

### 2.2 Anti‐Fungal Activity

**FIGURE 2:**
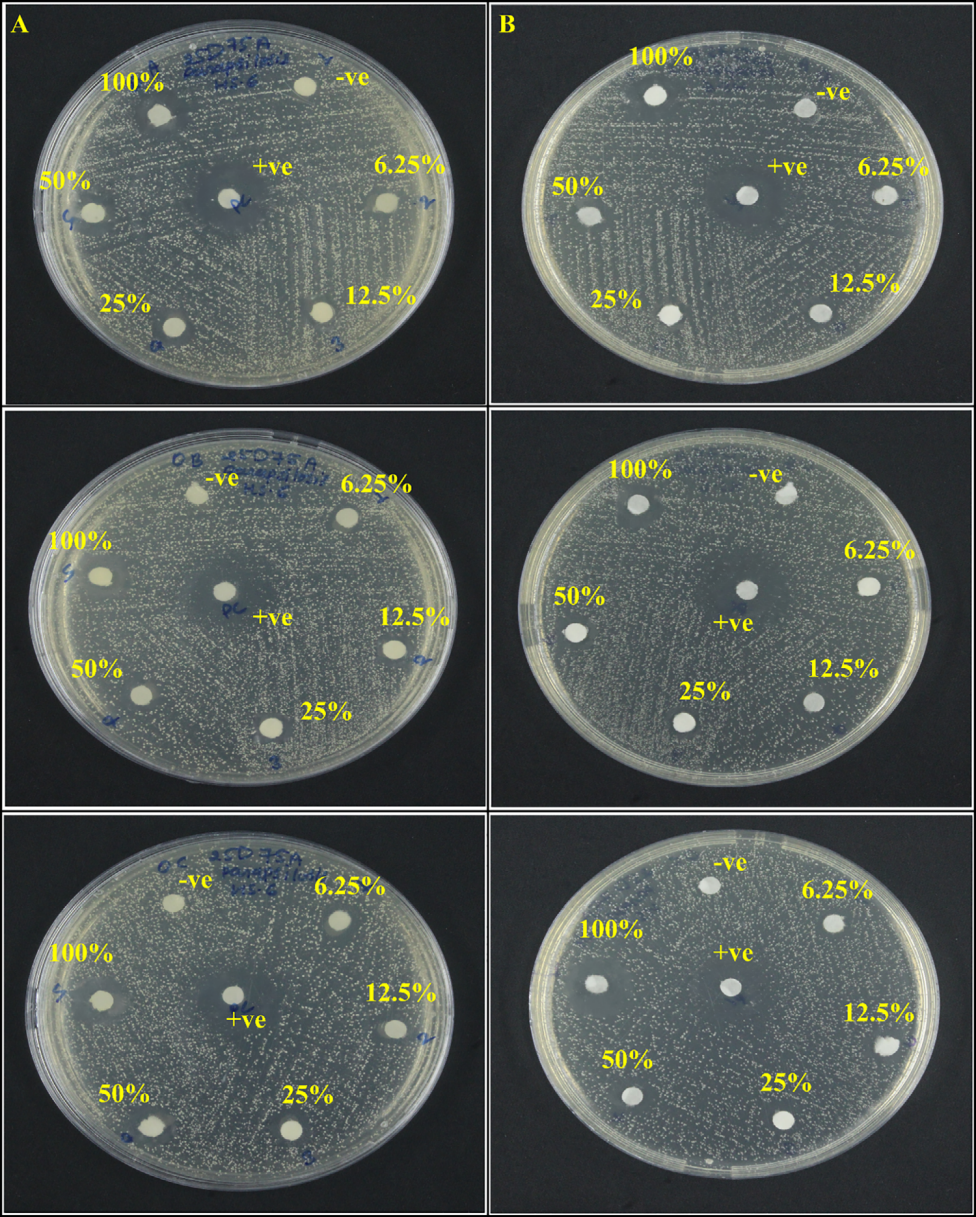
Zones of inhibition produced by HSEO against (A). *C. parapsilosis* and (B). *C. tropicalis*, showing HSEO concentrations of 6.25%, 12.5%, 25%, 50%, and 100% (v/v), along with positive (+ve) and negative (−ve) controls. Each panel represents three experimental replicates.

The antifungal activity of *H. spicatum* essential oil (HSEO) against *C. parapsilosis* demonstrated a clear and well‐defined concentration‐dependent pattern (Table 3). All experiments were conducted in triplicate (*n* = 3), and the results are presented as mean ± standard deviation to ensure accuracy, reliability, and reproducibility of the experimental findings. No inhibition was observed in the negative control (0.00 ± 0.00 mm), confirming the absence of any solvent‐related antifungal effect and validating the integrity of the assay conditions. At 6.25% (v/v), HSEO produced an inhibition zone of 5.20 ± 0.10 mm, indicating the Lowest tested concentration producing inhibition. The antifungal activity increased progressively and consistently with increasing concentration, reaching 6.40 ± 0.17 mm at 12.5%, 7.80 ± 0.26 mm at 25%, and 9.20 ± 0.35 mm at 50%. The maximum inhibition was recorded at 100% (v/v), with a zone diameter of 11.00 ± 0.53 mm, reflecting the strongest antifungal response under the tested conditions (Table S2). In comparison, the reference drug fluconazole (20 µg/disc) produced a significantly larger inhibition zone of 15.50 ± 0.46 mm (Figure 2A). One‐way ANOVA revealed highly significant differences among treatments (*p* < 0.0001).

**TABLE 3:** Antifungal activity of HSEO against *C. parapsilosis* and *C. tropicalis*, expressed as zones of inhibition (mm; mean ± SD; *n* = 3).

S. no.	Treatment	Concentration	*C. parapsilosis* (mm)	*C. tropicalis* (mm)
1	Negative control	—	0.00 ± 0.00	0.00 ± 0.00
2	HSEO	6.25% (v/v)	5.20 ± 0.10	4.80 ± 0.17
3	HSEO	12.5% (v/v)	6.40 ± 0.17	5.90 ± 0.35
4	HSEO	25% (v/v)	7.80 ± 0.26	7.10 ± 0.26
5	HSEO	50% (v/v)	9.20 ± 0.35	8.60 ± 0.40
6	HSEO	100% (v/v)	11.00 ± 0.53	10.40 ± 0.36
7	Fluconazole (positive control)	20 µg/disc	15.50 ± 0.46	16.53 ± 0.15
8	Lowest tested concentration producing inhibition		6.25% (v/v)	6.25% (v/v)
9	ANOVA	*F* value	566.84	235.1
10		*p* value	<0.0001	<0.0001
11		Remark	Highly significant	Highly significant

*HSEO: *H. spicatum* essential oil.

Similarly, HSEO showed a clear concentration‐dependent antifungal activity against *C. tropicalis* under identical experimental conditions. The negative control showed no inhibition (0.00 ± 0.00 mm), further confirming the absence of any background interference. At 6.25% (v/v), the inhibition zone measured 4.80 ± 0.17 mm, which increased steadily to 5.90 ± 0.35 mm at 12.5%, 7.10 ± 0.26 mm at 25%, and 8.60 ± 0.40 mm at 50%, demonstrating a gradual enhancement in antifungal potency with increasing concentration. The highest concentration (100% v/v) resulted in a zone of 10.40 ± 0.36 mm, representing the maximum observed inhibition for this strain (Table S3). Fluconazole demonstrated a stronger antifungal effect, producing a zone of 16.53 ± 0.15 mm under the same assay conditions. Statistical analysis using one‐way ANOVA again indicated highly significant differences among treatments (*p* < 0.0001). Overall, HSEO displayed marked and concentration‐responsive antifungal activity against both *Candida* strains, with comparatively greater relative activity observed against *C. parapsilosis* than *C. tropicalis*, particularly at the highest tested concentration.

### 2.3 Homology Modeling

**FIGURE 3:**
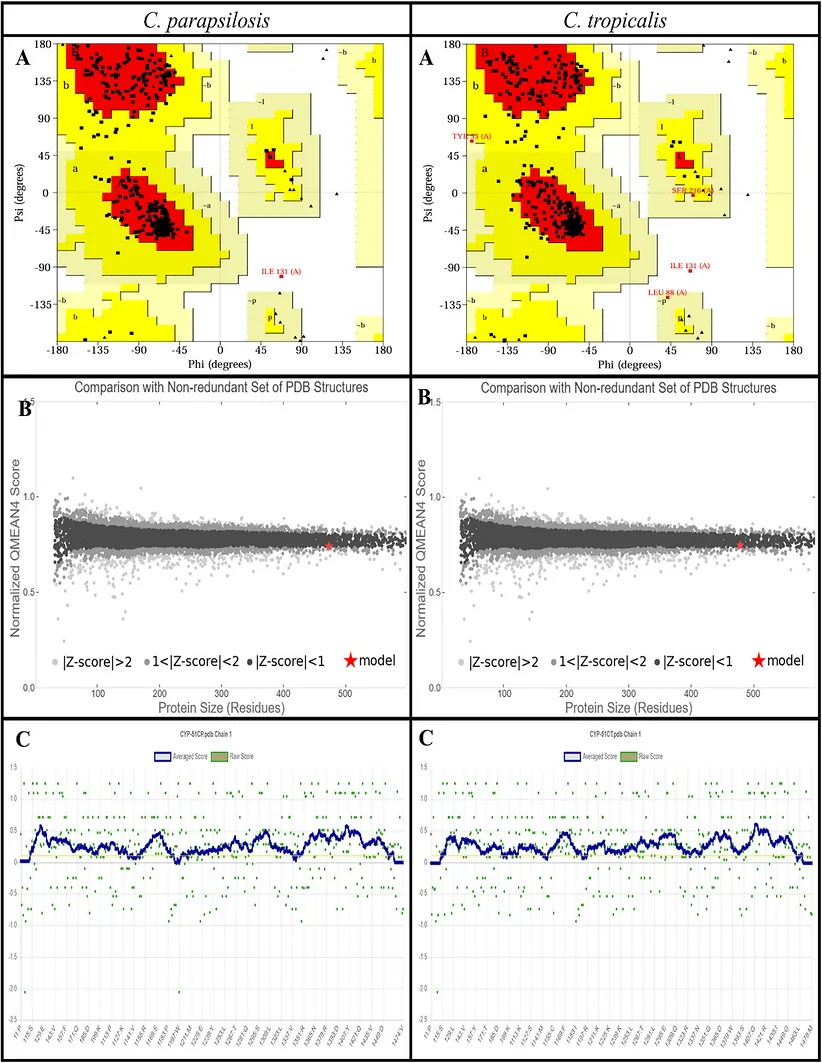
Validation of homology‐modeled CYP51 from *C. parapsilosis* and *C. tropicalis*. (A) Ramachandran plots showing residues predominantly in favored regions. (B) QMEAN4 comparison with non‐redundant PDB structures (red star indicates the model). (C) Verify3D profile.

The target proteins of *C. parapsilosis* and *C. tropicalis* were constructed using homology modelling based on the *C. albicans* CYP‐51 crystal structure (PDB ID 5TZ1) as the template. The sequence identity between the template and target proteins was 76.53% and 82.73%, respectively, indicating a high level of homology suitable for reliable model generation. Ramachandran plot analysis revealed that over 99% of residues were located in favored and additionally allowed regions, with only 0.2% in disallowed regions, indicating excellent stereochemical quality of the predicted structures. As shown in Figure 3A, most residues were distributed within the α‐helical region, followed by β‐sheet conformations, with only a small fraction located in the left‐handed α‐helix region. The QMEAN4 scores were −0.98 and −0.51 for *C. parapsilosis* and *C. tropicalis*, respectively. These values, being close to zero, suggest that the overall model quality is comparable to experimentally resolved structures of similar size (Figure 3B). The GMQE scores for both models were 0.83, indicating high expected modelling accuracy. Verify3D analysis demonstrated that 89.24% (*C. parapsilosis*) and 89.77% (*C. tropicalis*) of residues had an averaged 3D‐1D score ≥ 0.2, exceeding the acceptable threshold of 80%, thereby supporting the structural plausibility of the models (Figure 3C). The ERRAT quality factors were 93.819% and 92.979%, respectively, indicating satisfactory non‐bonded interaction profiles. Furthermore, the MolProbity scores of 1.36 and 1.53 suggest good overall geometry with minimal steric clashes. All validation parameters are summarized in Table 4.

**TABLE 4:** Homology models of CYP51 from *C. parapsilosis* and *C. tropicalis* generated for subsequent analyses.

S. no.	Parameters	CYP‐51	
		*C. parapsilosis*	*C. tropicalis*
1	Template (PDB ID)	CYP‐51 *C. albicans* (5TZ1)	CYP‐51 *C. albicans* (5TZ1)
2	No. of amino acids	523	527
3	Sequence identity (%)	76.53	83.72
4	Residue sequence coverage	49–522	49–527
5	GMQE	0.83	0.83
6	QMEAN Z‐score	−0.98	−0.51
7	ERRAT (%)	93.819	92.979
8	Verify3D (%)	89.24	89.77
9	MolProbity Score	1.36	1.53
10	Ramachandran favored (%)	91.3	89.4
11	Ramachandran allowed (%)	8.4	9.7
12	Ramachandran disallowed (%)	0.2	0.2

### 2.4 Molecular Docking and Redocking

All 29 essential oil compounds were docked against the haem‐free CYP51 of *C. parapsilosis* and *C. tropicalis*; the docking scores ranged from −7.6 to −5.7 kcal/mol and from −7.9 to −5.5 kcal/mol, respectively, suggesting strong interactions between active site residues and essential oil compounds. The top five compounds with the lowest docking score, with their interacting amino acids, were provided in Table 5. The redocking study revealed that the RMSD values between the native ligand and the corresponding docked poses were 1.56 and 1.78 Å for the *C. parapsilosis* haem‐free CYP51 and *C. tropicalis* haem‐free CYP51 ligands, respectively.

**TABLE 5:** Binding interactions of *H. spicatum* essential oil compounds with haem‐free CYP51 from *C. parapsilosis* and *C. tropicalis* obtained through molecular docking.

S. no.	Compound	*C. parapsilosis* haem‐free CYP51		Compound	With *C. tropicalis* haem‐free CYP51	
		Docking score (kcal/mol)	Interacting amino acid		Docking score (kcal/mol)	Interacting amino acid
1	cis‐γ‐Bisabolene	−7.6	Ser502, Met503, Pro231, Phe381, His378, Tyr64, Phe234, Ser379, Leu121, Leu377, Tyr118, Phe132, Ile380, Thr122	cis‐γ‐Bisabolene	−7.9	Leu376, Tyr118, Leu121, Phe380, Phe233, Tyr64, Leu87, His377, Pro230, Ser378, Met508, Thr122, Tyr132
2	γ‐Eudesmol	−7.4	Val504, Phe229, Met503, Leu377, Ser379, Phe381, Pro231, Phe234, Leu121, Tyr118, Thr122, Phe132, Phe126	γ‐Muurolene	−7.5	Met508, Tyr118, Pro230, His377, Ser378, Leu376, Phe228, Val509, Tyr132, Thr122, Leu121
3	γ‐Muurolene	−7.2	Phe132, Ala114, His463, Arg382, Ile380, Leu377, Leu121, Tyr118, Met503, Thr122, Phe229, Phe126, Ile131	δ‐Cadinene	−7.4	Tyr118, Thr122, Leu121, Thr311, His310, Val509, Leu376, Phe228, Gly307, Phe126, Met508, Tyr132, Ile131
4	β‐Guaiene	−7.1	Phe132, Tyr118, Leu377, Thr122, Phe229, Phe126, Gly308, Ile131	α‐Eudesmol	−7.2	Phe228, Ser378, Ile379, Phe380, Met508, Leu121, Tyr118, Tyr132, Leu376, Thr122, Phe126, Gly307, Val509
5	Caryophyllene	−7.1	Leu121, Tyr118, Met503, Leu377, Phe229, Thr312, Gly308, Ile131, Phe126, Phe132, Thr122, Phe234	Caryophyllene	−7.1	Ser378, Leu121, Tyr118, Tyr132, Thr122, Met508, Phe228, Leu376
6	Fluconazole (reference drug)	−6.7	Leu121, Phe234, Leu377, Tyr118, Met503, Phe132, Thr122, Ile131, Phe126, Phe229, Ile380, His468, Ser379, Phe381	Fluconazole (Reference drug)	−7.1	Tyr132, Arg381, His468, Ile379, Tyr118, Arg469, Cys470

Molecular docking analysis of *C. parapsilosis* haem‐free CYP51 revealed that cis‐γ‐Bisabolene exhibited the lowest docking score (−7.6 kcal/mol), interacting with 14 active site residues, including Ser503, Leu121, and Phe132. The observed interaction distances (3.92–5.06 Å) suggest predominantly hydrophobic contacts such as *π*–sigma, alkyl, and *π*‒alkyl interactions (Figure 4A). γ‐Eudesmol showed a docking energy of −7.4 kcal/mol and interacted with 13 active site residues, mainly Ser503, Phe234, Leu121, and Tyr118, with an average interaction distance of 3.70 Å, indicating π‐alkyl interactions. γ‐Muurolene demonstrated a docking score of −7.2 kcal/mol and formed interactions with 13 residues, primarily through *π*–alkyl and *π*–sigma contacts (Figure 4B). Both β‐Guaiene and Caryophyllene exhibited docking scores of −7.1 kcal/mol, interacting with 8 and 12 amino acid residues, respectively, with interaction distances ranging from 4.68 to 5.24 Å, predominantly through *π*–alkyl interactions (Figure 4C,D). The ligand cis‐γ‐Bisabolene exhibited stable binding within the active pocket through multiple hydrophobic and *π*–alkyl interactions with residues such as Phe381, Phe234, Leu121, Tyr118, and Phe132. These interactions, observed at distances of approximately 3.9–5.2 Å, suggest favorable stabilization of the ligand within the protein binding site (Figure 4E). The reference drug Fluconazole also showed a docking score of −7.1 kcal/mol, interacting with 14 active site residues within 4.40–5.40 Å, involving *π*–*π* stacked, *π*–alkyl, and *π*–*π* T‐shaped interactions (Figure 4F).

FIGURE 4Molecular docking of *H. spicatum* essential oil compounds with haem‐free CYP51 of *C. parapsilosis*. Left (3D binding pose); Right (2D interaction diagram). (A) β‐guaiene, (B) δ‐cadinene, (C) Ledol, (D) Cubebol, (E) cis‐γ‐bisabolene, and (F) fluconazole (reference drug).

**Figure:**
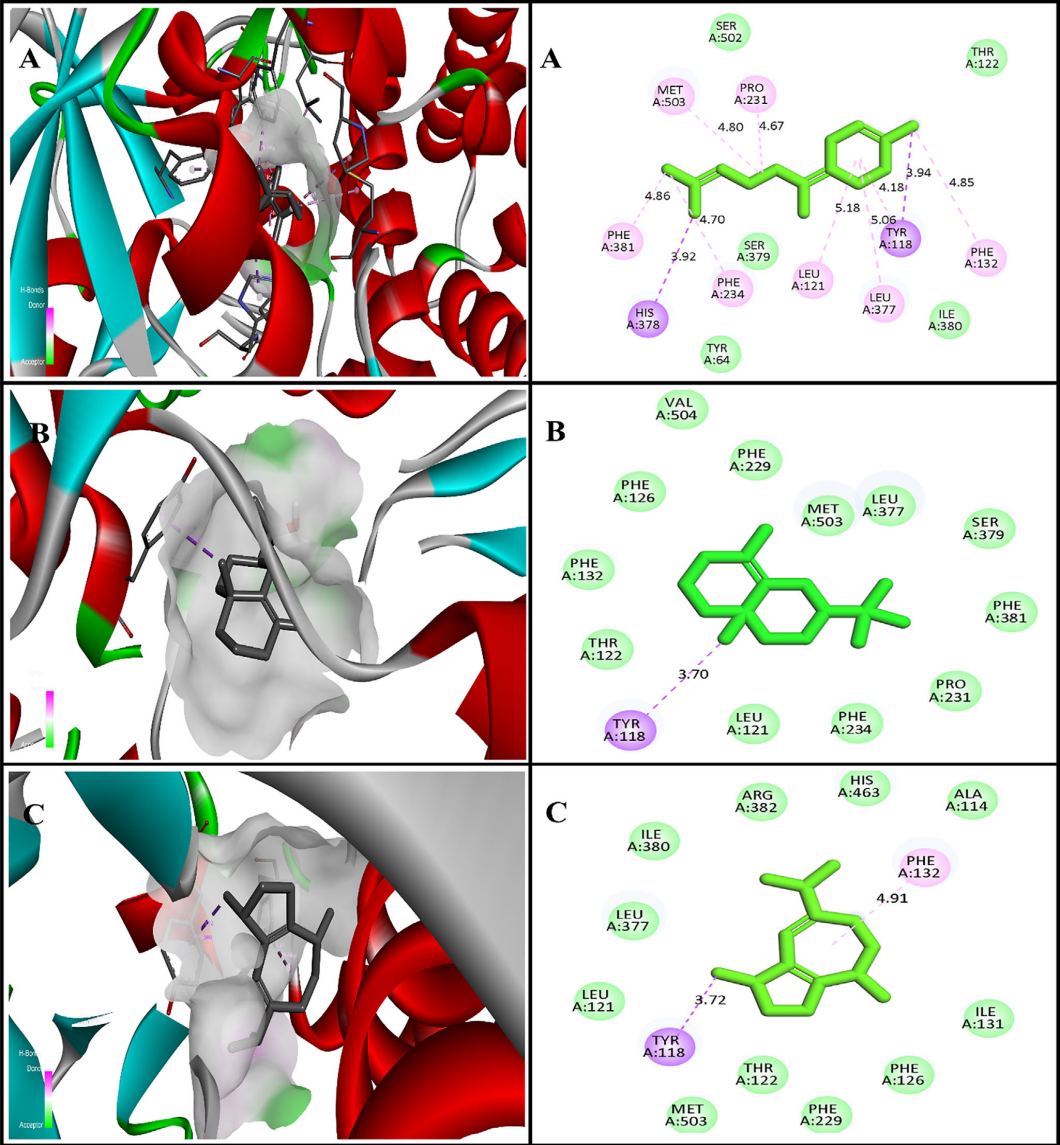

**Figure:**
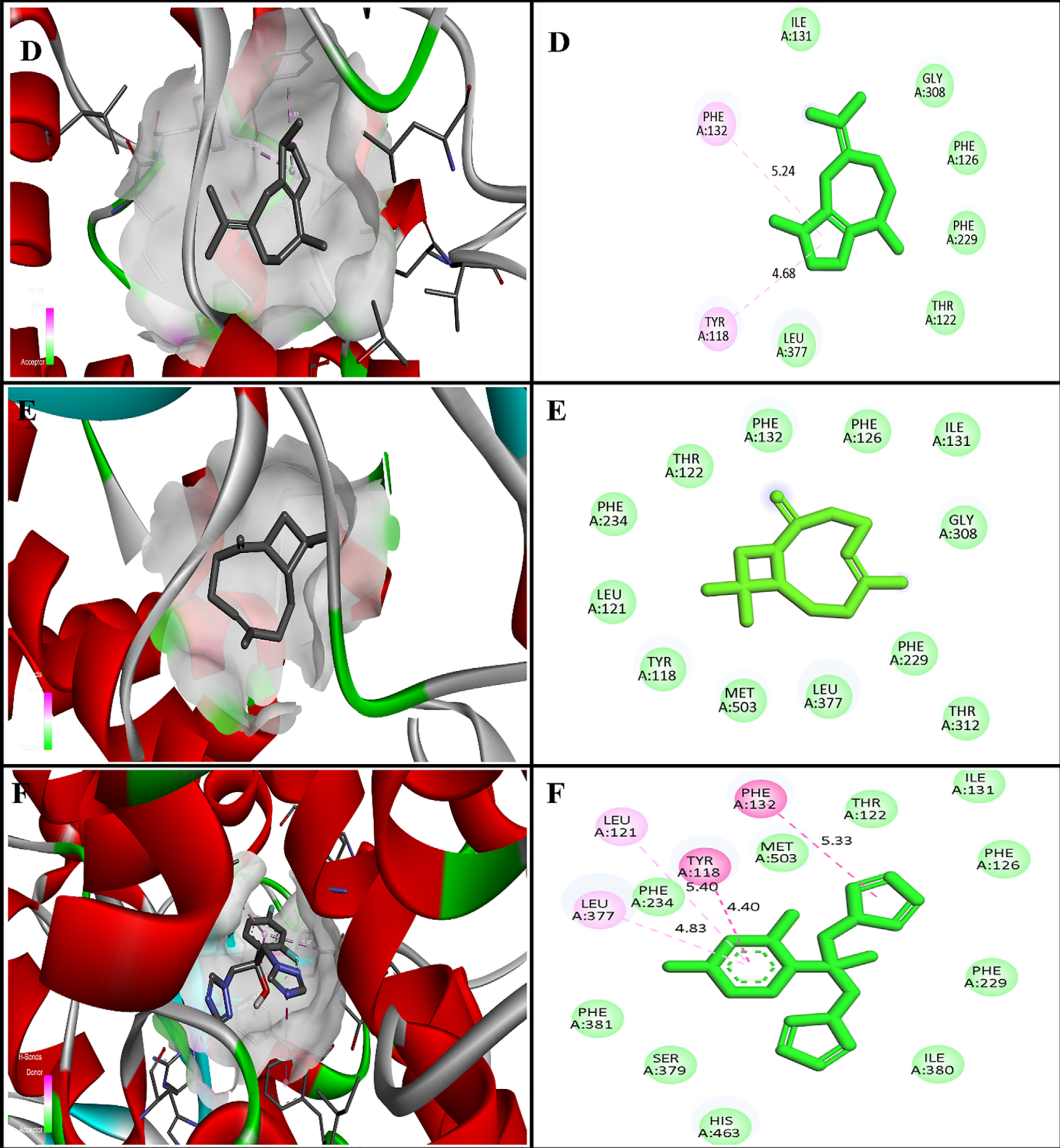

Docking analysis against *C. tropicalis* haem‐free CYP51 showed that cis‐γ‐Bisabolene exhibited the highest binding affinity with a docking score of −7.9 kcal/mol, interacting with 13 active site residues, including Leu376, Tyr118, Phe380, and Phe233. The interaction distances ranged from 3.69 to 5.42 Å (Figure 5A). γ‐Muurolene demonstrated a docking score of −7.5 kcal/mol and interacted with 11 amino acid residues, including Met508, Pro230, and Leu121, primarily through *π*–alkyl interactions with distances between 4.30 and 5.35 Å (Figure 5B). δ‐Cadinene showed a docking score of −7.4 kcal/mol, forming interactions with 13 residues such as Leu121, Phe125, Met508, and Phe228. The interactions included *π*–sigma, *π*–alkyl, and alkyl contacts with bond lengths ranging from 3.87 to 5.38 Å (Figure 5C). α‐Eudesmol exhibited a docking score of −7.2 kcal/mol and interacted with 13 residues, including Phe228, Phe126, Gly307, and Val509, predominantly through alkyl and *π*–alkyl interactions (5.04–5.44 Å) (Figure 5D). Caryophyllene showed a docking score of −7.1 kcal/mol, interacting with eight active site residues such as Ser378, Tyr132, and Met508, mainly through alkyl interactions (4.98–5.14 Å) (Figure 5E). The reference drug Fluconazole also demonstrated a docking score of −7.1 kcal/mol and interacted with seven amino acid residues, including Arg469, Tyr132, and Cys470. The interaction distances ranged from 2.50 to 5.54 Å and involved hydrogen‐bonding, *π*–alkyl, and carbon–hydrogen bond interactions (Figure 5F).

FIGURE 5Molecular docking of *H. spicatum* essential oil compounds with haem‐free CYP51 of *C. tropicalis*. Left (3D binding pose); Right (2D interaction diagram). (A) β‐Guaiene, (B) caryophyllene, (C) Ledol, (D) cis‐γ‐bisabolene, (E) γ‐muurolene, and (F) fluconazole (reference drug).

**Figure:**
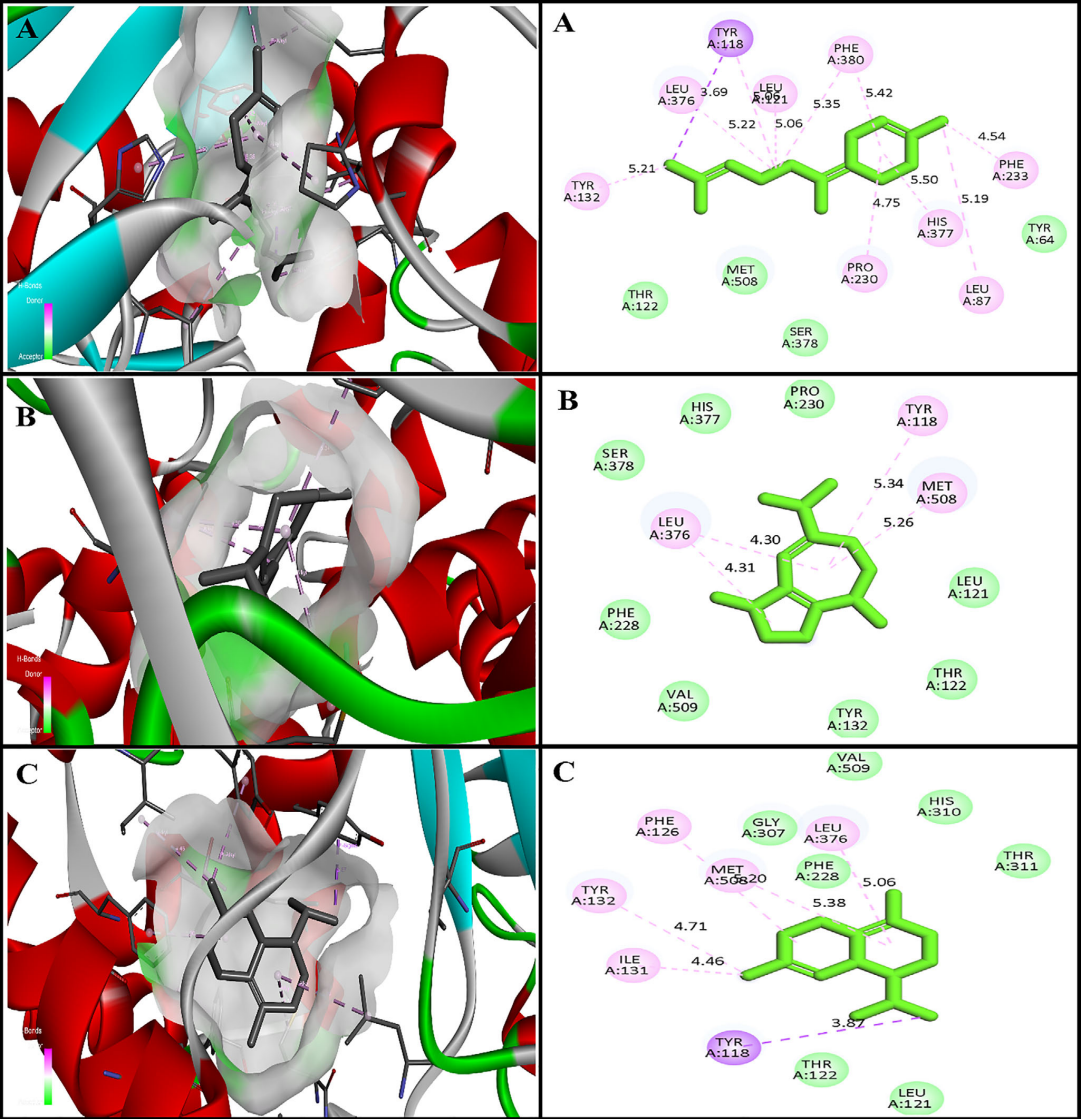

**Figure:**
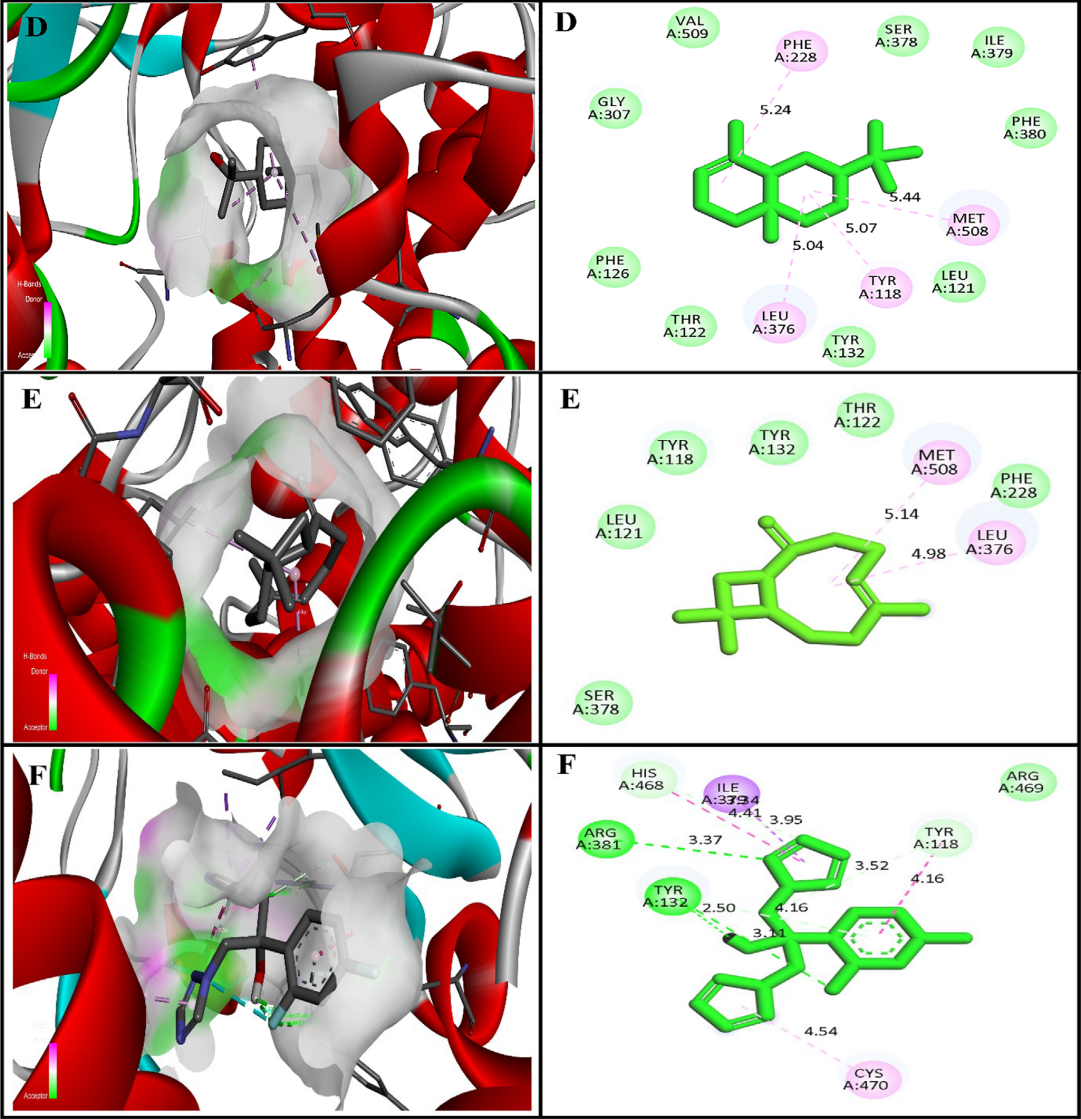

### 2.5 ADMET

The ADMET predictions provided a preliminary assessment of the pharmacokinetic and toxicity‐related properties of cis‐γ‐bisabolene in comparison with fluconazole (Table 6). Although its predicted aqueous solubility was lower, the predicted intestinal absorption value (> 94%) suggests favorable absorption potential. The absence of predicted P‐glycoprotein substrate behavior suggests a lower likelihood of efflux‐mediated transport. cis‐γ‐Bisabolene was also predicted to have limited inhibitory activity toward the evaluated CYP450 isoforms, suggesting a potentially lower risk of CYP‐mediated drug interactions than fluconazole. Compliance with Lipinski's Rule of Five further supports its predicted drug‐like properties. The predicted absence of hepatotoxicity and AMES mutagenicity provides preliminary computational evidence of a favorable safety profile. However, these predictions do not establish clinical safety, oral suitability, or suitability for human consumption and require experimental validation.

**TABLE 6:** Predicted ADMET properties of the computationally prioritized compound and reference drugs.

S. no.	Parameters	cis‐γ‐Bisabolene	Fluconazole (reference drug)
1	Water solubility (log mol/L)	−6.188	−3.293
2	Intestinal absorption human (%)	94.493	94.960
3	P‐gp substrate	No	No
4	CYP1A2 inhibitor	No	Yes
5	CYP2C19 inhibitor	No	No
6	CYP2C9 inhibitor	No	No
7	CYP2D6 inhibitor	No	No
8	CYP3A4 inhibitor	No	No
9	Lipinski	Yes	Yes
10	Hepatotoxicity	No	Yes
11	AMES Toxicity	No	No

### 2.6 MD Simulation

**FIGURE 6:**
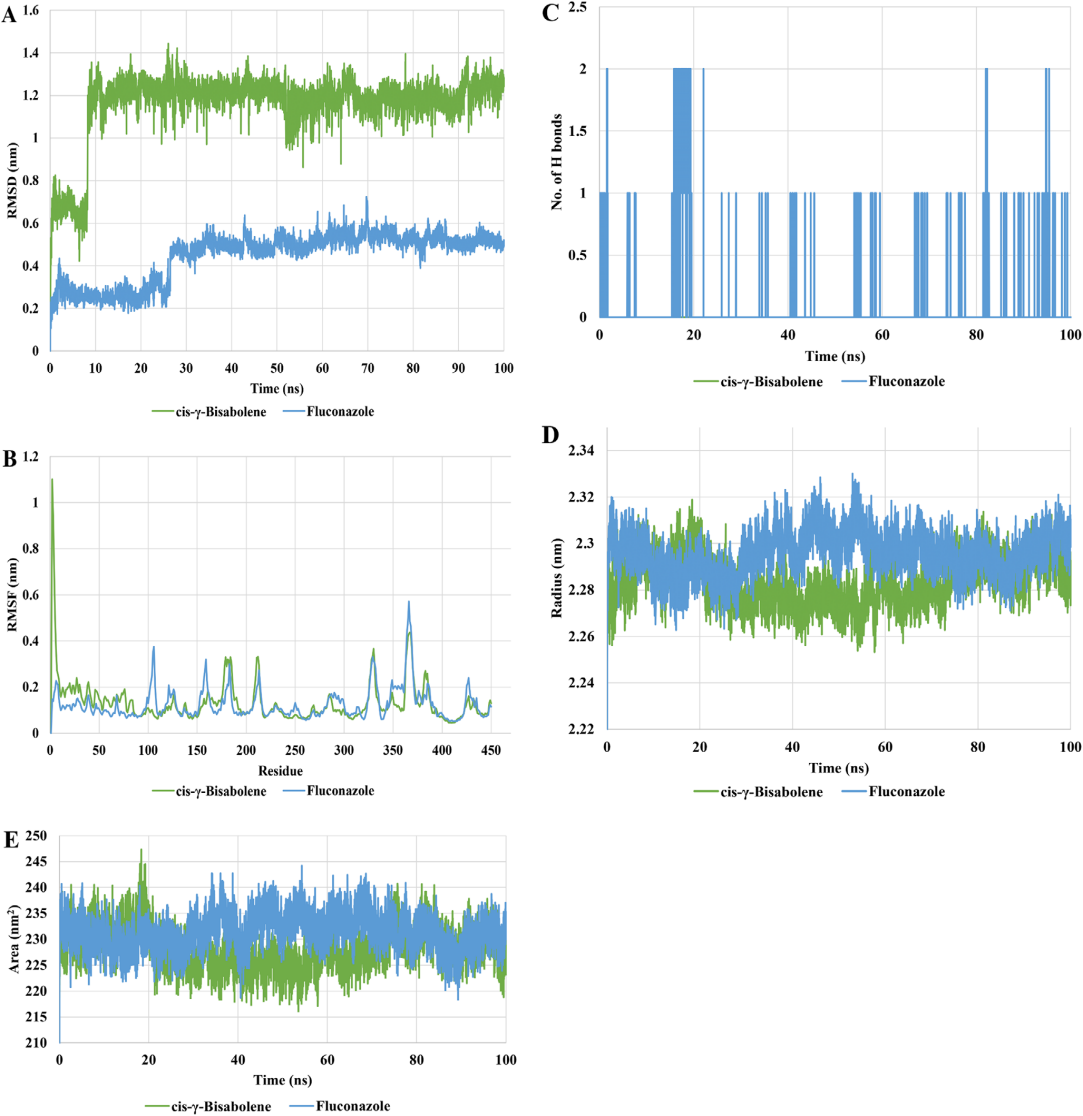
Molecular dynamics simulation analysis of *C. parapsilosis* haem‐free CYP51 in complex with cis‐γ‐bisabolene and fluconazole. (A) RMSD, (B) RMSF, (C) hydrogen bond analysis, (D) radius of gyration (*R*_g_), and (E) SASA.

The molecular dynamics simulation of *C. parapsilosis* haem‐free CYP51 complexes demonstrated stable structural behavior for both ligands. The RMSD values of the backbone atoms stabilized after the initial equilibration phase and remained within ∼1.20–1.35 nm for the cis‐γ‐bisabolene complex and ∼0.6–0.7 nm for the fluconazole complex, indicating overall conformational stability throughout the trajectory. RMSF analysis showed that most residues fluctuated below ∼0.15–0.25 nm, with comparatively higher deviations observed in flexible loop regions, while active‐site residues remained relatively stable in both systems. Hydrogen bond analysis revealed that the fluconazole‐haem‐free CYP51 complex consistently maintained 1–2 hydrogen bonds during the simulation, whereas cis‐γ‐bisabolene did not form hydrogen bonds due to the absence of hydrogen‐bond donor and acceptor groups in its structure. The radius of gyration (*R*_g_) for the cis‐γ‐bisabolene complex ranged between ∼2.26–2.32 nm, whereas the fluconazole complex fluctuated between ∼2.30–2.33 nm, indicating comparatively greater compactness in the cis‐γ‐bisabolene‐bound system. Similarly, SASA values for cis‐γ‐bisabolene varied from ∼225–245 nm^2^, while fluconazole exhibited slightly higher values of ∼235–245 nm^2^, reflecting relatively reduced solvent exposure and maintained structural compactness in the cis‐γ‐bisabolene complex. Collectively, these results indicate stable complex formation of cis‐γ‐bisabolene with *C. parapsilosis* haem‐free CYP51, predominantly stabilized through hydrophobic interactions. All values were presented in Figure 6A–E.

**FIGURE 7:**
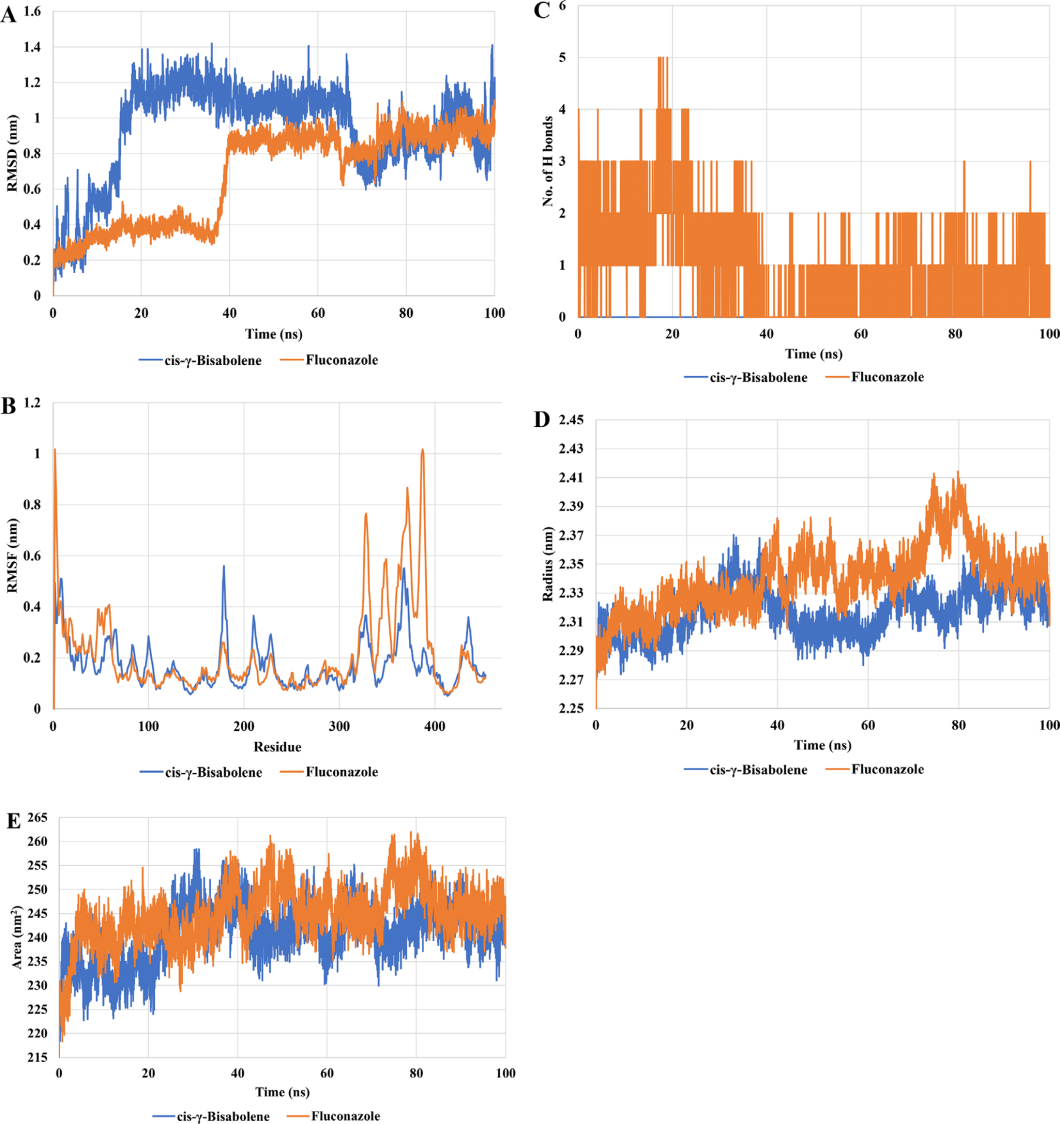
Molecular dynamics simulation analysis of *C. tropicalis* haem‐free CYP51 in complex with cis‐γ‐bisabolene and fluconazole. (A) RMSD, (B) RMSF, (C) hydrogen bond analysis, (D) radius of gyration (*R*_g_), and (E) SASA.

The molecular dynamics simulation of *C. tropicalis* haem‐free CYP51 complexes demonstrated stable structural behavior for both ligands. The RMSD values of the backbone atoms stabilized after the initial equilibration phase and remained within ∼1.00–1.20 nm for the cis‐γ‐bisabolene complex and ∼0.8–1.10 nm for the fluconazole complex, indicating overall conformational stability throughout the trajectory. RMSF analysis showed that most residues fluctuated below ∼0.15–0.25 nm, with comparatively higher deviations observed in flexible loop regions. Hydrogen bond analysis revealed that the fluconazole CYP51 complex consistently maintained 1–5 hydrogen bonds during the simulation, whereas cis‐γ‐bisabolene did not form hydrogen bonds due to the absence of hydrogen bond donor and acceptor groups in its structure. The radius of gyration (*R*_g_) for the cis‐γ‐bisabolene complex ranged between ∼2.29 and 2.34 nm, whereas the fluconazole complex fluctuated between ∼2.30 and 2.40 nm, indicating comparatively greater compactness in the cis‐γ‐bisabolene‐bound system. Similarly, SASA values for cis‐γ‐bisabolene varied from ∼225 to 250 nm^2^, while fluconazole exhibited slightly higher values of ∼235–260 nm^2^, reflecting relatively reduced solvent exposure and maintained structural compactness in the cis‐γ‐bisabolene complex. Collectively, these results indicate stable complex formation of cis‐γ‐bisabolene with *C. tropicalis* haem‐free CYP51, predominantly stabilized through hydrophobic interactions. All values were presented in Figure 7A–E.

### 2.7 Binding Free Energy Analysis (MM‐PBSA)

The binding free energy components of cis‐γ‐bisabolene and fluconazole with haem‐free CYP51 of *C. parapsilosis* and *C. tropicalis* were calculated using MM‐PBSA analysis (Table 7). For haem‐free CYP51 of *C. parapsilosis*, cis‐γ‐bisabolene exhibited a van der Waals energy (ΔE_vdW) of −27.63 kJ/mol, while fluconazole showed −28.76 kJ/mol. The electrostatic energy (ΔE_elec) was minimal for cis‐γ‐bisabolene (−0.27 kJ/mol) compared to fluconazole (−2.69 kJ/mol). The polar solvation energy (ΔG_polar) contributed unfavorably for both ligands, with values of 5.64 kJ/mol for cis‐γ‐bisabolene and 12.59 kJ/mol for fluconazole. The nonpolar solvation energy (ΔG_nonpolar) was −21.82 kJ/mol for cis‐γ‐bisabolene and −19.93 kJ/mol for fluconazole. The total binding free energy (ΔG_bind) was −6.02 kJ/mol for cis‐γ‐bisabolene, whereas fluconazole showed a slightly positive value of 0.98 kJ/mol.

**TABLE 7:** MM/PBSA binding free‐energy components of haem‐free CYP51–Cis‐γ‐bisabolene and CYP51–Fluconazole complexes.

		Haem‐free CYP51 of *C. parapsilosis*		Haem‐free CYP51 of *C. tropicalis*	
S. no.	Parameters	cis‐γ‐Bisabolene	Fluconazole (Reference drug)	cis‐γ‐Bisabolene	Fluconazole (Reference drug)
1	△E_vdW	−27.63	−28.76	−29.60	−30.11
2	△E_elec	−0.27	−2.69	−0.60	−4.34
3	△G_polar	5.64	12.59	7.52	15.72
4	△G_nonpolar	−21.82	−19.93	−24.18	−21.09
5	△G_bind	−6.02	0.98	−8.05	0.66

*△E_vdW: van der Waals Energy; △E_elec: Electrostatic energy; △G_polar: Polar Solvatoin Energy; △G_nonpolar: Non‐polar Solvation Energy; △G_bind: Binding Free Energy.

For haem‐free CYP51 of *C. tropicalis*, cis‐γ‐bisabolene demonstrated a ΔE_vdW of −29.60 kJ/mol, while fluconazole exhibited −30.11 kJ/mol. The electrostatic contribution was −0.60 kJ/mol for cis‐γ‐bisabolene and −4.34 kJ/mol for fluconazole. The polar solvation energy was 7.52 kJ/mol for cis‐γ‐bisabolene and 15.72 kJ/mol for fluconazole. The ΔG_nonpolar values were −24.18 and −21.09 kJ/mol for cis‐γ‐bisabolene and fluconazole, respectively. Notably, the overall binding free energy (ΔG_bind) was −8.05 kJ/mol for cis‐γ‐bisabolene, while fluconazole again showed a slightly positive value of 0.66 kJ/mol.

### 2.8 DFT Analysis

**FIGURE 8:**
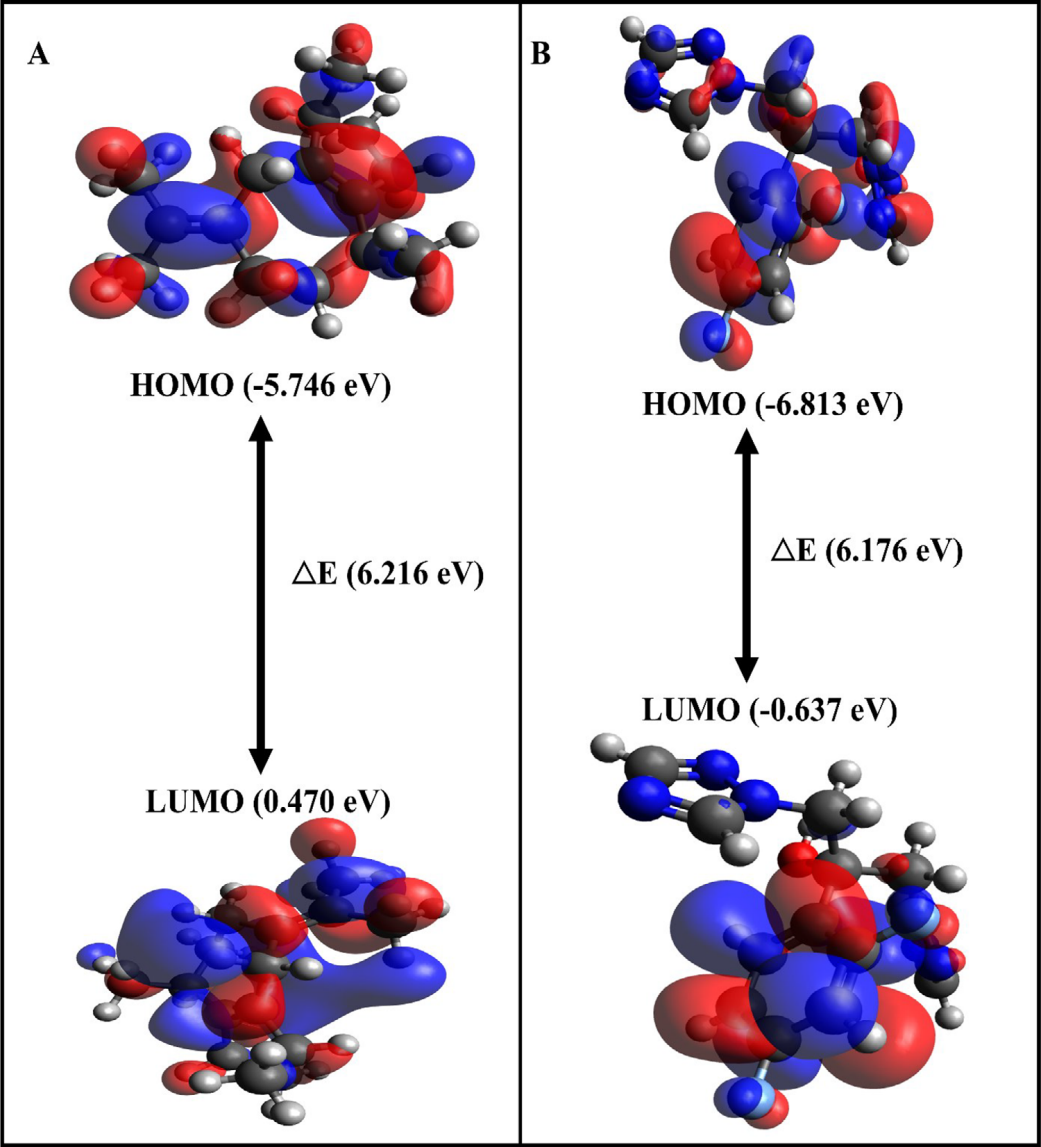
HOMO and LUMO molecular orbitals of (A) cis‐γ‐bisabolene and (B) fluconazole (reference drug).

The frontier molecular orbital (FMO) energies and global reactivity descriptors of cis‐γ‐bisabolene (final hit compound) were compared with the reference drug fluconazole (Table 8). The calculated HOMO and LUMO energies of cis‐γ‐bisabolene were −5.746 and 0.470 eV, respectively, yielding an energy gap (Δ*E*) of 6.216 eV (Figure 8A). Fluconazole exhibited a very similar energy gap of 6.176 eV. The extremely small difference in Δ*E* (0.040 eV) indicates that both compounds possess nearly identical kinetic stability (Figure 8B). However, cis‐γ‐bisabolene shows a marginally higher energy gap. A comparable observation was noted for global hardness (*η*), where cis‐γ‐bisabolene (3.108 eV) displayed a slightly higher value than fluconazole (3.088 eV). However, the difference (0.020 eV) is minimal, suggesting that both molecules exhibit similar resistance to charge transfer and comparable electronic rigidity. More noticeable variation was observed in electronegativity (*χ*) and chemical potential (*μ*). Fluconazole (*χ* = 3.725 eV) showed higher electronegativity than cis‐γ‐bisabolene (*χ* = 2.638 eV). Similarly, the electrophilicity index (*ω*) demonstrated a clearer distinction between the two compounds. Fluconazole exhibited a higher electrophilicity value (2.247 eV), suggesting stronger electron‐accepting (electrophilic) character, whereas cis‐γ‐bisabolene showed a lower electrophilicity index (1.119 eV), indicating comparatively weaker electrophilic behavior.

**TABLE 8:** HOMO–LUMO energies and calculated global reactivity descriptors of cis‐γ‐bisabolene and fluconazole.

S. no.	Parameters	cis‐γ‐Bisabolene	Fluconazole	Remark
1	HOMO (eV)	−5.746	−6.813	Higher HOMO indicates better electron‐donating ability (cis‐γ‐bisabolene is slightly higher)
2	LUMO (eV)	0.470	−0.637	Lower LUMO suggests stronger electron‐accepting ability (fluconazole is lower)
3	Energy gap △*E* (eV)	6.216	6.176	Comparable energy gap indicating similar kinetic stability
4	Global hardness *η* (eV)	3.108	3.088	Similar resistance to charge transfer
5	Global softness *S* (eV^−1^)	0.161	0.324	Fluconazole shows higher electron density flexibility
6	Electronegativity *χ* (eV)	2.638	3.725	Fluconazole has a stronger electron‐attracting tendency
7	Chemical potential *μ* (eV)	−2.638	−3.725	More negative value indicates greater stability (fluconazole)
8	Electrophilic index *ω* (eV)	1.119	2.247	Fluconazole exhibits stronger electrophilic character

## 3 Discussion

The increasing incidence of invasive candidiasis caused by *C. tropicalis* and *C. parapsilosis* poses a significant clinical challenge, particularly due to the rising emergence of antifungal resistance [19]. These non‐*a*lbicans *Candida* species are increasingly associated with bloodstream infections and immunocompromised conditions, thereby necessitating the exploration of alternative and plant‐derived natural therapeutic strategies [1]. Among plant‐based products, essential oils have attracted considerable attention owing to their diverse volatile bioactive constituents and well‐documented antimicrobial properties. In the present study, GC‐MS analysis of *H. spicatum* essential oil revealed the presence of 29 phytoconstituents belonging to monoterpene and sesquiterpene classes. τ‐Cadinol was identified as the predominant constituent, followed by 1,8‐cineole and other OS. Similar chemical profiles have been reported previously, indicating consistency in the phytochemical composition of the species across studies [20]. OS such as *τ*‐cadinol are known to exhibit antifungal activity, potentially through disruption of fungal cell membrane integrity, alteration of permeability, and interference with essential metabolic processes [21, 22].

The antifungal evaluation of HSEO demonstrated a clear concentration‐dependent response against both *Candida* species. The lowest tested concentration producing inhibition was observed at 6.25% (v/v), indicating the lowest concentration capable of producing measurable inhibition. At 100% (v/v), inhibition zones of 11.00 ± 0.53 mm for *C. parapsilosis* and 10.40 ± 0.36 mm for *C. tropicalis* were recorded. Although these values were lower than those obtained for fluconazole, they reflect moderate antifungal activity of the essential oil. Comparable inhibition zones (∼10 mm) have been reported in previous studies evaluating plant essential oils against *C. albicans*, supporting the reproducibility of such antifungal effects [23]. The comparatively reduced inhibition relative to fluconazole may be attributed to the lack of structural optimization for specific enzyme targeting [16]. Unlike synthetic azoles, which are designed to selectively inhibit CYP51, essential oils exert their effects through multi‐component, multi‐target mechanisms [12].

CYP51 is a validated antifungal target, as its inhibition disrupts ergosterol synthesis and fungal membrane integrity [10]. The three‐dimensional structure of CYP51 was generated using the SWISS‐MODEL server based on a suitable template with high sequence identity. The constructed model demonstrated satisfactory stereochemical quality, as 89% of residues were positioned in the favored regions, 9.7% in the allowed regions, and fewer than 0.2% in the disallowed regions of the Ramachandran plot, indicating its suitability for subsequent molecular docking studies. The QMEAN Z‐Score was also in an acceptable range [24], which means the quality of the model is good. The ERRAT score was also between 90+%, suggesting overall non‐bonded interactions as reported [25]. The Verify3D score is also above the threshold value of 80%, indicating good compatibility between the amino acid sequence and the modelled three‐dimensional structure [26]. All 29 phytoconstituents were docked against haem‐free CYP51 of *C. tropicalis* and *C. parapsilosis*. cis‐γ‐Bisabolene exhibited the lowest binding energy against both enzymes, interacting with 13 active‐site residues through predominantly hydrophobic contacts, indicating stable binding. Fluconazole showed slightly lower binding energies (−7.1 and −6.7 kcal/mol) compared to cis‐γ‐bisabolene. All the ligands are mainly interacting with van der Waals, pi–alkyl, alkyl bonds, which suggesting ligand binding is majorly dominated by hydrophobic interaction. The redocking RMSD values were below the widely accepted cutoff of 2.0 Å, thereby validating the accuracy and reliability of the employed docking protocol [27].

The ADMET analysis suggests that cis‐γ‐bisabolene possesses a favorable pharmacokinetic profile comparable to fluconazole. Although its predicted aqueous solubility was lower, the high intestinal absorption value (>94%) indicates good oral bioavailability potential [28]. The absence of P‐glycoprotein substrate behavior further suggests reduced susceptibility to efflux‐mediated elimination [29]. Notably, cis‐γ‐bisabolene was predicted to be non‐inhibitory toward major CYP450 isoforms, a lower risk of drug–drug interactions compared to fluconazole, particularly with respect to CYP1A2 inhibition [30]. Compliance with Lipinski's Rule of Five supports its drug‐likeness characteristics. Importantly, the compound was predicted to be non‐hepatotoxic and AMES‐negative, indicating a favorable preliminary safety profile. Collectively, these predictions highlight the potential of cis‐γ‐bisabolene as a pharmacologically viable antifungal candidate.

MD simulation was conducted to evaluate the dynamic behavior of the protein–ligand complex under near‐physiological conditions. RMSD, RMSF, hydrogen bonds, Rg, and SASA were analyzed to assess stability, compactness, and flexibility. Both CYP51‐ligand complexes showed an initial RMSD increase (20–30 ns) followed by stabilization. Cis‐γ‐bisabolene remained within the active‐site cavity and showed movement toward the deeper hydrophobic region during the simulation. This positional adjustment may reflect conformational adaptation of the ligand within the binding cavity [31]. RMSF analysis did not reveal any unusual fluctuation patterns. Higher flexibility was primarily observed at the N‐ and C‐terminal regions, which is typical for proteins, along with moderate fluctuations in a few residues that may be involved in ligand binding or local conformational adjustments [32]. Hydrogen bond analysis showed that fluconazole maintained 3–5 stable hydrogen bonds throughout the simulation, indicating persistent polar interactions within the active site of Lanosterol 14‐alpha demethylase. In contrast, cis‐γ‐bisabolene, lacking hydrogen‐bond donor and acceptor groups, did not form classical hydrogen bonds and is likely stabilized primarily through hydrophobic and van der Waals interactions [33]. The *R*g and SASA remained stable for both complexes throughout the simulation, with average *R*g values around ∼2.3 nm and SASA ranging between 220–260 nm^2^; similar values of SASA and *R*g were also observed in the study of a 496 amino‐acid protein [34]. These values fall within the expected range for a moderately sized protein, indicating the absence of significant structural compaction or unfolding during the simulation [34, 35].

MM‐PBSA offers valuable quantitative insight into receptor‐ligand binding affinity, while also decomposing the total binding energy into its major contributing components, including van der Waals, polar (electrostatic), and nonpolar interactions [36]. Previous studies have indicated that MM‐PBSA‐derived binding free energies may range from negative to slightly positive values depending on solvation effects and methodological limitations; one study also found MM‐PBSA energy ranging from −187 to +62 kJ/mol [36]. Therefore, the slightly positive ΔG_bind values observed for fluconazole should be interpreted comparatively rather than absolutely. Energy decomposition analysis revealed that binding in both haem‐free CYP51 systems was primarily driven by van der Waals and nonpolar contributions, whereas electrostatic interactions were comparatively minor. Notably, fluconazole exhibited substantially higher polar solvation penalties, which offset its favorable electrostatic interactions. These findings support the structural characteristic of CYP51 possessing a predominantly hydrophobic active‐site cavity, where ligand stabilization appears to be largely governed by hydrophobic interactions. Structural studies on human CYP51 demonstrated that inhibitors primarily occupy a hydrophobic binding pocket and are stabilized mainly through van der Waals and hydrophobic interactions [37]. The stronger nonpolar contribution observed for cis‐γ‐bisabolene further suggests that nonpolar ligands may be favorably accommodated within the CYP51 binding pocket.

Quantum chemical calculations were performed at 310 K in the presence of water (implicit solvent model) to mimic biological conditions and to evaluate the intrinsic chemical reactivity, kinetic stability, and electronegativity of the individual compounds [38]. The calculated HOMO–LUMO energy gaps of cis‐γ‐bisabolene (6.216 eV) and fluconazole (6.176 eV) fall within the typical range reported for stable drug‐like organic molecules (generally ∼4–8 eV) [39]. The calculated global hardness (*η* ≈ 3.1 eV) is consistent with a chemically stable framework. According to conceptual DFT principles, increased hardness reflects resistance to charge transfer and structural perturbation [40]. Such hardness values suggest resistance to abrupt charge redistribution while still permitting controlled interaction with biological targets. The electronegativity (*χ*) values observed for both compounds (2.6–3.7 eV) also lie within the typical range reported for pharmacologically active heterocyclic and hydrocarbon‐based drugs [41], indicating a relatively stronger tendency to attract electrons during molecular interactions. Fluconazole's higher electronegativity indicates a stronger tendency to attract electrons, which may enhance polar and coordinate interactions within the CYP51 active site. Similarly, electrophilicity index (*ω*) values between ∼1–3 eV are generally associated with moderate electrophilic character suitable for biological interactions without excessive reactivity; a similar range was also observed in the study of [42]. Overall, the descriptor ranges for cis‐γ‐bisabolene align well with those of established drug‐like compounds, indicating appropriate electronic stability and reactivity for potential biological activity.

Although cis‐γ‐bisabolene was identified as a major computationally prioritized constituent with potential haem‐free CYP51‐binding activity, its specific contribution to the antifungal activity of *H. spicatum* essential oil remains computationally predicted, as the purified compound was not isolated or independently evaluated in the present study. Experimental assessment of purified cis‐γ‐bisabolene is therefore necessary to determine its individual antifungal activity and validate its proposed contribution to CYP51 inhibition. The present study also addresses an existing gap in the literature by providing experimental evidence of the antifungal activity of *H. spicatum* essential oil against *Candida* species. Furthermore, the computational analysis identified cis‐γ‐bisabolene as a putative bioactive constituent that exhibited favorable interactions with the fungal target CYP51, providing a basis for its further experimental investigation.

## 4 Conclusion

The present study provides an integrated experimental and computational evaluation of the essential oil derived from the rhizomes of *H. spicatum*. GC‐MS analysis identified 29 phytochemical constituents, highlighting its chemical complexity. In vitro antifungal assays showed good to moderate activity against *Candida* species, with inhibition zones of ∼10–11 mm, supporting its biological relevance. High‐quality homology models of CYP51 were developed and used for docking and simulation studies. Docking revealed that cis‐γ‐bisabolene exhibited the lowest binding energy against *C. parapsilosis* haem‐free CYP51 and showed stronger predicted affinity toward *C. tropicalis* haem‐free CYP51. ADMET analysis provided preliminary predictions of its pharmacokinetic and toxicity‐related properties. MD simulations confirmed structural stability of the complexes, and MM‐PBSA analysis supported comparatively stronger binding of cis‐γ‐bisabolene. DFT results suggested comparable kinetic stability to fluconazole, although the reference drug showed relatively stronger electrophilic character. Overall, *H. spicatum* essential oil demonstrates promising antifungal potential against *Candida* species, likely involving CYP51 inhibition. Future studies may explore cis‐γ‐bisabolene as a potential lead compound for the development of therapeutic agents against *Candida* species, including antifungal‐resistant strains.

## 5 Experimental Section

The experimental and analytical procedures carried out in this study are described in detail below.

### 5.1 Plant Collection and Sample Preparation

A wild sample of *H. spicatum* rhizome was collected from Pithoragarh (29°35′ N, 80°13′ E), Uttarakhand, India, in December, 2025. The plant material was authenticated at the Botanical Survey of India (BSI), Dehradun, India, and a voucher specimen was submitted (Voucher No. 1862). For further study, freshly collected rhizomes were washed thoroughly with running water to remove soil and debris.

### 5.2 Essential Oil Extraction

Freshly obtained rhizomes were hydro‐distilled to extract essential oil using a Clevenger‐type apparatus in accordance with method [43]. The rhizomes were precisely weighed and then hydro‐distilled for 5 h. Then the essential oil was purified by redistillation. The collected essential oil was dehydrated using anhydrous sodium sulfate (Na_2_SO_4_) and stored in sealed vials at 4°C until further analysis [44]. The weight of the fresh rhizomes was used to determine the percentage content of the essential oils that were recovered.

$$\mathrm{Oil}\;\mathrm{yield}\;\%=\frac{\mathrm{Volume}\;\mathrm{of}\;\mathrm{extracted}\;\mathrm{EO}\;(\mathrm{mL})}{\mathrm{Fresh}\;\mathrm{weight}\;\mathrm{of}\;\mathrm{plant}\;\mathrm{sample}\;(g)}\;\times \;100$$

### 5.3 GC and GC‐MS

Gas chromatography (GC) analysis of the essential oil was performed using a Nucon 5765 gas chromatograph equipped with a dual flame ionization detector (FID) and Rtx‐5 and CP‐Wax 52 CB fused silica columns. Nitrogen and hydrogen were used as carrier gases at a flow rate of 1.0 mL/min. The oven temperature was programmed from 60 to 230°C at 4°C/min. Injector and detector temperatures were maintained at 210°C and 220°C, respectively, with a split ratio of 1:30. GC‐MS analysis was carried out using an Agilent 8890/5977B system fitted with a DB‐5MS column. The oven temperature was programmed from 50°C to 300°C at 5°C/min using helium as the carrier gas. Mass spectra were recorded in electron impact (EI) mode at 70 eV over a mass range of 30–550 amu. Identification of compounds was based on comparison of retention index (RI), calculated relative to a homologous series of n‐alkanes (C_9_–C_24_), and matching with NIST (20.L) and literature [45]. GC‐MS analysis was performed as a single analytical run without replicates.

### 5.4 Fungus Culture and Maintenance

Cultures of two pathogenic fungi, *C. parapsilosis* (MTCC 998) and *C. tropicalis* (MTCC 1000), were obtained from the Microbial Type Culture Collection and Gene Bank (MTCC), Chandigarh, India. The strains were maintained on Sabouraud Dextrose Agar (SDA) and subcultured periodically at 37°C. For antifungal assays, inoculum was prepared in Sabouraud Dextrose Broth (SDB) and adjusted to 0.5 McFarland standard, corresponding to approximately 1.5 × 10^8^ CFU/mL [46].

### 5.5 Antifungal Activity

Antifungal activity of HSEO was evaluated using the agar disc diffusion (Kirby–Bauer) method following Clinical and Laboratory Standards Institute (CLSI) guidelines [47, 48]. Sterile discs (5 mm) prepared from Whatman No. 1 filter paper were impregnated with 5 µL of different concentrations (0%–100%) of HSEO. Dimethyl sulfoxide (DMSO) was used as the solvent and served as the vehicle control. Fluconazole (20 µg/disc) was used as the positive control. SDA plates were inoculated by spreading 100 µL of standardized fungal suspension, followed by placement of the prepared discs. The plates were incubated at 37°C for 48 h. The diameter of the inhibition zones (ZOI) around the discs was measured and recorded in millimeters (mm). All experiments were performed in triplicate to ensure reliability, precision, and reproducibility of results.

### 5.6 Statistical Analysis

Statistical analysis was performed using PAST 4.03 software. One‐way ANOVA was used to assess differences among the experimental groups, with statistical significance set at *p* < 0.05. Three experimental replicates were used for the antifungal activity measurements.

### 5.7 Homology Modelling

The amino acid sequences of lanosterol 14α‐demethylase (CYP51) from *C. parapsilosis* (NCBI accession no. XNX61098.1) and *C. tropicalis* (NCBI accession no. ATO93827.1) were retrieved in FASTA format from the NCBI (https://www.ncbi.nlm.nih.gov/) protein database. Homology modeling was performed using the SWISS‐MODEL (https://swissmodel.expasy.org/) server under default parameters. Template structures were automatically selected based on sequence identity, query coverage, and Global Model Quality Estimation (GMQE) scores. The best‐predicted models were selected for further analysis based on template similarity and the QMEAN scoring function to ensure structural reliability. Model validation was conducted to assess stereochemical quality and structural consistency. The reliability of the predicted models was evaluated using Ramachandran plot analysis, ERRAT score, and the VERIFY3D program using the SAVESv6.1 server (https://saves.mbi.ucla.edu/).

### 5.8 Molecular Docking and Redocking

The three‐dimensional structures of selected phytochemicals were retrieved from the PubChem database in SDF format (https://pubchem.ncbi.nlm.nih.gov/). Ligand structures were retrieved from the PubChem database in SDF format and converted to PDBQT format using Open Babel. Hydrogen atoms were added during ligand preparation, followed by energy minimization using the Universal Force Field (UFF) with 100 optimization steps [49]. The minimized structures were then converted to PDBQT format using AutoDock Tools. The receptor protein was energy‐minimized using the steepest descent algorithm for 500 steps in UCSF Chimera to relieve steric clashes (https://www.cgl.ucsf.edu/chimerax/). The receptor proteins were prepared using AutoDock Tools by adding polar hydrogen atoms and assigning Gasteiger charges, followed by conversion to PDBQT format. The active site of the receptor was taken as the same as that occupied by the native ligand. The grid centers were set at *x* = 3.5239, *y* = 0.7412, *z* = −0.4850 for *C. parapsilosis* haem‐free CYP51 and *x* = 3.3997, *y* = 0.9008, *z* = −0.5623 for *C. tropicalis* haem‐free CYP51, with a grid size of 20 Å along the *x*, *y*, and *z* axes. Molecular docking was performed using the Linux version of AutoDock Vina (version 1.2) [50]. Molecular docking was performed using AutoDock Vina with an exhaustiveness value of 16 and an energy range of 4 kcal/mol, generating a maximum of nine binding poses for each ligand. The pose with the most negative predicted binding affinity was selected for subsequent interaction analysis and molecular dynamics simulations. The protocol was validated by redocking the native ligand into the active site and comparing the predicted pose with its original crystallographic orientation. During homology modelling of both CYP51 proteins using SWISS‐MODEL, oteseconazole was retained in the generated models as the co‐crystallized ligand. The ligand was subsequently re‐docked at its original binding position to validate the docking protocol.

### 5.9 ADMET

The ADMET predictions were used to evaluate the drug‐likeness, safety, and potential toxicity risks associated with the selected phytocompounds. The pharmacokinetic and toxicity profiles of the selected phytocompounds were evaluated using the online servers pkCSM and ADMETlab 3.0 [51, 52]. The canonical SMILES of each compound were used as input for prediction. The assessed parameters included water solubility, human intestinal absorption (HIA), P‐glycoprotein (P‐gp) substrate prediction, cytochrome P450 (CYP) enzyme interactions, Lipinski's rule of five compliance [53], hepatotoxicity, and AMES toxicity. The predicted ADMET properties were analyzed to evaluate the drug‐likeness and safety profile of the compounds for human oral use.

### 5.10 Molecular Dynamics Simulation

MD simulations were performed using GROMACS version 2025.3 (https://www.gromacs.org/). All molecular dynamics simulations were performed using the CHARMM36 force field implemented in GROMACS [54]. The ligand parametrization was carried out using the SwissParam web (https://www.swissparam.ch/). The protein–ligand complexes were solvated in a triclinic simulation box using the TIP3P water model [55]. Appropriate counterions were added to neutralize the system. Energy minimization was carried out prior to equilibration. Following energy minimization, the systems were equilibrated under NVT and NPT ensembles for 100 ps each at 300 K, with NPT equilibration performed at 1 bar. Long‐range electrostatic interactions were treated using the Particle Mesh Ewald (PME) method, with 1.0 nm cutoffs for short‐range interactions. Production MD simulations were then conducted for 100 ns with a time step of 2 fs. A single independent MD simulation was performed for each protein–ligand complex. Post‐simulation trajectory analyses were performed to evaluate structural stability and dynamics, including root mean square deviation (RMSD), root mean square fluctuation (RMSF), hydrogen‐bond analysis (H‐bond), radius of gyration (*R*g), and solvent‐accessible surface area (SASA) [38].

### 5.11 MMPBSA Binding Free Energy Calculation

The binding free energy of the protein–ligand complexes was calculated using the gmx_MMPBSA tool based on the Molecular Mechanics/Poisson–Boltzmann Surface Area (MM‐PBSA) method [56]. The analysis was performed on the last 30 ns of the MD trajectory from 70–100 ns, from which 300 uniformly distributed frames were extracted for calculation. The calculations used an ionic strength of 0.150 M, with solute and solvent dielectric constants of 2.0 and 80.0, respectively. Formal uncertainty and convergence analyses were not performed.

### 5.12 DFT Calculations

Density functional theory (DFT) calculations were performed using ORCA 6.0 [57]. Geometry optimization and frequency calculations were carried out using the B3LYP functional with D3BJ dispersion correction and the def2‐SVP basis set [58, 59]. Calculations were performed at 310 K to mimic physiological temperature [38]. The solvent effect of water was included using the CPCM model (dielectric constant *ε* = 78.3553). Global reactivity descriptors including chemical hardness (*η*), softness (*S*), electronegativity (*χ*), chemical potential (*μ*), and electrophilicity index (*ω*) were calculated [60].

## 6 Limitations

The CYP51 homology models generated using SWISS‐MODEL lacked the haem cofactor, which may affect predicted ligand orientation, interaction patterns, binding energies, molecular dynamics behavior, and MM‐PBSA estimates. Thus, the computational findings should be considered exploratory predictions of potential binding within a haem‐free model rather than evidence of physiological CYP51 inhibition. Additionally, GC–MS was performed as a single analytical run; therefore, the reproducibility of the essential‐oil chemical profile and relative abundance of constituents, including cis‐γ‐bisabolene, could not be assessed. Experimental validation using haem‐containing CYP51 models, replicate GC–MS analyses, and standardized MIC/MFC assays would strengthen these findings.

## Author Contributions

**Preeti Verma**: Sample collection, GC‐MS analysis, antifungal activity assessment, and research design. **Devesh Singh Baral**: Manuscript drafting, formal analysis, in silico pipeline execution, methodology, and research design. **Rakesh Verma**: Supervision, methodology, statistical analysis, review, and editing.

## Conflicts of Interest

The authors declare no conflicts of interest.

## Supporting information




**Supporting File 1**: cbdv71742‐sup‐0001‐SuppMat.docx

## Data Availability

The data that support the findings of this study are available in the Supporting Information of this article.
