# Supplementary material for: Antifungal Activity of Hedychium spicatum Essential Oil Against Candidemia‐Causing Candida tropicalis and Candida parapsilosis: In Vitro Evaluation and Computational Identification of Cis‐γ‐Bisabolene as a Putative CYP51‐Binding Constituent
Source: Chem Biodivers. 2026 Sep 27;23(9):e71742. doi: 10.1002/cbdv.71742 (PMC13616462; doi:10.1002/cbdv.71742)
Supplement: Supplementary file 1 [file CBDV-23-e71742-s001.docx]

**Antifungal Activity of *Hedychium spicatum* Essential Oil against Candidemia-Causing *Candida tropicalis* and *Candida parapsilosis*: In Vitro Evaluation and Computational Identification of cis-γ-Bisabolene as a Putative CYP51-Binding Constituent**

Preeti Verma^1^, Devesh Singh Baral^2^ & Rakesh Verma*^2^

1. Department of Chemistry, PG College Kapkot (263632), SSJ University Almora, Uttarakhand, India
2. Department of Zoology, SSJ University, L.S.M. Campus, Pithoragarh (262502), Uttarakhand, India

[Corresponding author*] Email: [Rv.pith@gmail.com](mailto:Rv.pith@gmail.com) Contact no. +91-9897368158 ORCID: 0009-0002-2891-419X

**Table S1:** Raw chromatogram Data

| **No.** | **Peak Name** | **Retention time**  **min** | **Area**  **pA*min** | **Height**  **pA** | **Relative Area**  **%** | **Relative Height**  **%** | **Amount**  **n.a.** |
| --- | --- | --- | --- | --- | --- | --- | --- |
| 1. |  | 4.963 | 0.305 | 10.055 | 0.13 | 0.27 | n.a. |
| 2. | α-Pinene | 5.128 | 2.555 | 81.707 | 1.05 | 2.23 | n.a. |
| 3. |  | 5.440 | 0.123 | 3.104 | 0.05 | 0.08 | n.a. |
| 4. |  | 5.888 | 1.048 | 30.278 | 0.43 | 0.83 | n.a. |
| 5. | Sabinene | 6.005 | 8.210 | 234.221 | 3.38 | 6.40 | n.a. |
| 6. | β-Pinene | 6.162 | 0.324 | 9.113 | 0.13 | 0.25 | n.a. |
| 7. | Limonene | 6.837 | 3.073 | 64.513 | 1.26 | 1.76 | n.a. |
| 8. |  | 7.137 | 64.385 | 1303.913 | 26.49 | 35.62 | n.a. |
| 9. | 1,8-Cineole | 7.298 | 0.375 | 8.677 | 0.15 | 0.24 | n.a. |
| 10. | γ-Terpinene | 7.868 | 0.077 | 1.687 | 0.03 | 0.05 | n.a. |
| 11. |  | 8.185 | 10.280 | 222.596 | 4.23 | 6.08 | n.a. |
| 12. | Linalool | 8.950 | 0.082 | 1.198 | 0.03 | 0.03 | n.a. |
| 13. |  | 10.232 | 0.397 | 8.363 | 0.16 | 0.23 | n.a. |
| 14. | Trans-Sabinene hydrate | 11.003 | 0.255 | 5.259 | 0.11 | 0.14 | n.a. |
| 15. |  | 11.142 | 1.864 | 36.709 | 0.77 | 1.00 | n.a. |
| 16. | Terpinen-4-ol | 11.308 | 2.085 | 41.015 | 0.86 | 1.12 | n.a. |
| 17. | α-Terpineol | 11.702 | 2.085 | 41.015 | 0.86 | 1.12 | n.a. |
| 18. |  | 12.755 | 0.076 | 1.116 | 0.03 | 0.03 | n.a. |
| 19. |  | 13.212 | 0.162 | 2.584 | 0.07 | 0.07 | n.a. |
| 20. |  | 13.357 | 0.277 | 4.538 | 0.11 | 0.12 | n.a. |
| 21. |  | 15.952 | 0.176 | 2.755 | 0.07 | 0.08 | n.a. |
| 22. |  | 16.515 | 0.275 | 4.359 | 0.11 | 0.12 | n.a. |
| 23. |  | 16.652 | 0.031 | 0.490 | 0.01 | 0.01 | n.a. |
| 24. |  | 16.953 | 0.216 | 3.426 | 0.09 | 0.09 | n.a. |
| 25. |  | 17.353 | 1.025 | 12.673 | 0.42 | 0.35 | n.a. |
| 26. |  | 17.818 | 0.284 | 3.781 | 0.12 | 0.10 | n.a. |
| 27. |  | 18.192 | 0.081 | 1.462 | 0.03 | 0.04 | n.a. |
| 28. |  | 18.497 | 0.146 | 2.814 | 0.06 | 0.08 | n.a. |
| 29. | Isolongifelene | 18.723 | 1.964 | 30.201 | 0.81 | 0.82 | n.a. |
| 30. |  | 18.990 | 0.114 | 1.805 | 0.05 | 0.05 | n.a. |
| 31. | β-Caryophyllene | 19.645 | 1.012 | 17.199 | 0.42 | 0.47 | n.a. |
| 32. |  | 19.738 | 1.874 | 26.949 | 0.77 | 0.74 | n.a. |
| 33. | β-Acoradiene | 19.963 | 1.702 | 25.009 | 0.70 | 0.68 | n.a. |
| 34. |  | 20.373 | 1.601 | 23.908 | 0.66 | 0.65 | n.a. |
| 35. | γ-Muurolene | 20.543 | 0.737 | 10.641 | 0.30 | 0.29 | n.a. |
| 36. | Germacrene D | 20.968 | 4.781 | 39.332 | 1.97 | 1.07 | n.a. |
| 37. |  | 21.267 | 0.878 | 8.819 | 0.36 | 0.24 | n.a. |
| 38. |  | 21.498 | 2.657 | 33.303 | 1.09 | 1.61 | n.a. |
| 39. | cis-γ-Bisabolene | 21.753 | 12.740 | 203.719 | 5.24 | 5.56 | n.a. |
| 40. |  | 22.010 | 2.191 | 16.646 | 0.90 | 0.45 | n.a. |
| 41. |  | 22.150 | 1.949 | 17.398 | 0.80 | 0.48 | n.a. |
| 42. |  | 22.330 | 0.888 | 10.355 | 0.37 | 0.28 | n.a. |
| 43. | Cubebol | 22.517 | 2.314 | 35.853 | 0.95 | 0.98 | n.a. |
| 44. |  | 22.813 | 1.390 | 21.455 | 0.57 | 0.59 | n.a. |
| 45. |  | 23.048 | 0.095 | 1.273 | 0.04 | 0.03 | n.a. |
| 46. | δ-Cadinene | 23.313 | 4.677 | 79.992 | 1.92 | 2.19 | n.a. |
| 47. |  | 23.573 | 1.606 | 20.575 | 0.66 | 0.56 | n.a. |
| 48. |  | 23.868 | 0.101 | 1.431 | 0.04 | 0.04 | n.a. |
| 49. |  | 24.003 | 0.392 | 5.215 | 0.16 | 0.14 | n.a. |
| 50. |  | 24.142 | 1.051 | 14.851 | 0.43 | 0.41 | n.a. |
| 51. |  | 24.305 | 1.239 | 20.538 | 0.51 | 0.56 | n.a. |
| 52. | Elemol | 24.577 | 2.926 | 30.126 | 1.20 | 0.82 | n.a. |
| 53. |  | 24.775 | 2.927 | 21.578 | 1.20 | 0.59 | n.a. |
| 54. | γ-Eudesmol | 25.190 | 20.085 | 224.726 | 8.26 | 6.14 | n.a. |
| 55. | τ-Cadinol | 25.588 | 43.607 | 468.365 | 17.94 | 12.79 | n.a. |
| 56. |  | 25.957 | 0.884 | 6.884 | 0.36 | 0.19 | n.a. |
| 57. |  | 26.220 | 0.363 | 3.191 | 0.15 | 0.09 | n.a. |
| 58. |  | 26.363 | 0.340 | 4.770 | 0.14 | 0.13 | n.a. |
| 59. |  | 26.522 | 0.218 | 2.994 | 0.09 | 0.08 | n.a. |
| 60. |  | 26.845 | 0.659 | 7.484 | 0.27 | 0.20 | n.a. |
| 61. |  | 27.248 | 0.482 | 4.327 | 0.20 | 0.12 | n.a. |
| 62. |  | 27.595 | 0.084 | 1.418 | 0.03 | 0.04 | n.a. |
| 63. |  | 27.762 | 0.270 | 3.301 | 0.11 | 0.09 | n.a. |
| 64. | α-Eudesmol | 27.913 | 0.316 | 4.307 | 0.13 | 0.12 | n.a. |
| 65. |  | 28.177 | 0.114 | 0.979 | 0.05 | 0.03 | n.a. |
| 66. | α-Cadinol | 28.527 | 0.238 | 2.637 | 0.10 | 0.07 | n.a. |
| 67. |  | 28.742 | 0.078 | 1.057 | 0.03 | 0.03 | n.a. |
| 68. | 14-Hydroxy-(Z)-Caryophyllene | 29.368 | 1.607 | 5.365 | 0.66 | 0.15 | n.a. |
| 69. |  | 29.623 | 0.703 | 4.375 | 0.29 | 0.12 | n.a. |
| 70. |  | 29.907 | 0.100 | 1.263 | 0.04 | 0.03 | n.a. |
| 71. | Germacra 4(15),5,10(14) trien-1-α-ol | 31.228 | 19.338 | 54.227 | 7.96 | 1.48 | n.a. |
| 72. |  | 31.990 | 0.503 | 1.894 | 0.21 | 0.05 | n.a. |
| 73. |  | 33.057 | 0.471 | 3.953 | 0.19 | 0.11 | n.a. |
| 74. | 8-Cedren-13-ol | 33.350 | 0.030 | 0.429 | 0.01 | 0.01 | n.a. |
| 75. |  | 36.967 | 0.093 | 1.325 | 0.04 | 0.04 | n.a. |
| 76. |  | 37.968 | 0.308 | 1.823 | 0.13 | 0.05 | n.a. |
| Total | |  |  | 243.012 | 3660.798 | 100.00 | 100.00 |

**Table S2.** Raw zone of inhibition (ZOI) data for *C. parapsilosis* across all three replicates.

| **S.No.** | **Treatment** | **Rep1 (mm)** | **Rep2 (mm)** | **Rep3 (mm)** |
| --- | --- | --- | --- | --- |
| 1. | Negative control | 0.00 | 0.00 | 0.00 |
| 2. | 6.25% | 5.30 | 5.00 | 5.30 |
| 3. | 12.5% | 6.50 | 6.50 | 6.20 |
| 4. | 25% | 7.50 | 8.00 | 7.90 |
| 5. | 50% | 8.80 | 9.40 | 9.40 |
| 6. | 100% | 11.40 | 11.20 | 10.40 |
| 7. | Fluconazole | 15.60 | 15.00 | 15.90 |

**Table S3.** Raw zone of inhibition (ZOI) data for *C. tropicalis* across all three replicates.

| **S.No.** | **Treatment** | **Rep1 (mm)** | **Rep2 (mm)** | **Rep3 (mm)** |
| --- | --- | --- | --- | --- |
| 1. | Negative control | 0.00 | 0.00 | 0.00 |
| 2. | 6.25% | 4.60 | 4.70 | 5.10 |
| 3. | 12.5% | 5.40 | 6.10 | 6.20 |
| 4. | 25% | 6.60 | 6.90 | 8.40 |
| 5. | 50% | 8.10 | 9.00 | 8.70 |
| 6. | 100% | 10.20 | 10.70 | 10.30 |
| 7. | Fluconazole | 16.70 | 16.40 | 16.50 |

**Figure S1.** Chromatogram of HSEO
